# Lamellipodia-like actin networks in cells lacking WAVE regulatory complex

**DOI:** 10.1242/jcs.260364

**Published:** 2022-08-16

**Authors:** Frieda Kage, Hermann Döring, Magdalena Mietkowska, Matthias Schaks, Franziska Grüner, Stephanie Stahnke, Anika Steffen, Mathias Müsken, Theresia E. B. Stradal, Klemens Rottner

**Affiliations:** 1 Division of Molecular Cell Biology, Zoological Institute, Technische Universität Braunschweig, Spielmannstrasse 7, 38106 Braunschweig, Germany; 2 Department of Cell Biology, Helmholtz Centre for Infection Research, Inhoffenstrasse 7, 38124 Braunschweig, Germany; 3 Central Facility for Microscopy, Helmholtz Centre for Infection Research, 38124 Braunschweig, Germany; 4 Braunschweig Integrated Centre of Systems Biology (BRICS), 38106 Braunschweig, Germany

**Keywords:** Rac, Arp2/3 complex, VASP, N-WASP, Cdc42, Protrusion

## Abstract

Cell migration frequently involves the formation of lamellipodia induced by Rac GTPases activating WAVE regulatory complex (WRC) to drive Arp2/3 complex-dependent actin assembly. Previous genome editing studies in B16-F1 melanoma cells solidified the view of an essential, linear pathway employing the aforementioned components. Here, disruption of the WRC subunit Nap1 (encoded by *Nckap1*) and its paralog Hem1 (encoded by *Nckap1l*) followed by serum and growth factor stimulation, or active GTPase expression, revealed a pathway to formation of Arp2/3 complex-dependent lamellipodia-like structures (LLS) that requires both Rac and Cdc42 GTPases, but not WRC. These phenotypes were independent of the WRC subunit eliminated and coincided with the lack of recruitment of Ena/VASP family actin polymerases. Moreover, aside from Ena/VASP proteins, LLS contained all lamellipodial regulators tested, including cortactin (also known as CTTN), the Ena/VASP ligand lamellipodin (also known as RAPH1) and FMNL subfamily formins. Rac-dependent but WRC-independent actin remodeling could also be triggered in NIH 3T3 fibroblasts by growth factor (HGF) treatment or by gram-positive *Listeria monocytogenes* usurping HGF receptor signaling for host cell invasion. Taken together, our studies thus establish the existence of a signaling axis to Arp2/3 complex-dependent actin remodeling at the cell periphery that operates without WRC and Ena/VASP.

## Abstract (precis)

**Summary:** Rac-dependent actin remodeling can occur in the absence of WAVE regulatory complex, triggered by active Cdc42. WAVE regulatory complex-independent actin structures harbor Arp2/3 complex but not VASP.

## INTRODUCTION

Protrusions at the cell periphery are frequently driven by actin networks generated through the actin filament branching activity of the Arp2/3 complex (Rotty et al., 2013). With the exception of bundles containing parallel actin filaments, which are commonly referred to as filopodia and are likely driven by the Ena/VASP family of actin polymerases, formins or both (Gallop, 2020; Rottner et al., 2017), Arp2/3 complex-dependent actin networks give rise to various structures in distinct cell types. These include lamellipodia and ruffles that mediate migration or, presumably, micropinocytosis; the phagocytic cups of professional phagocytes; and the actin-dependent protuberances accompanying the induced entry of specific pathogens (Krause and Gautreau, 2014; Stradal and Schelhaas, 2018).

Actin remodeling in mammalian cells is largely regulated by small GTPases of the Rho family (Aspenström et al., 2004). In particular, members of the Rac subfamily (Rac1, Rac2 and Rac3) have emerged as key GTPases obligatory for membrane ruffling and lamellipodia formation in cell types ranging from metastasizing melanoma cells to mesenchymal fibroblasts, neurons and platelets (McCarty et al., 2005; Schaks et al., 2018; Steffen et al., 2013; Tahirovic et al., 2010). In early studies, there were conflicting ideas about precisely how Rac connects to WAVE subfamily members (WASF1, 2 or 3), key activators of the Arp2/3 complex in membrane ruffles and lamellipodia (Machesky and Insall, 1998). The most promising initial candidate was IRSp53 (also known as BAIAP2; Miki et al., 2000), which was also found to colocalize with WAVE at the edges of protruding lamellipodia (Nakagawa et al., 2003). However, even earlier studies had already described an alternative Rac interactor, termed Sra1 (specifically Rac-associated protein 1, also known as CYFIP1) (Eden et al., 2002; Kobayashi et al., 1998), which is now well established to constitute two interaction surfaces with Rac on heteropentameric WAVE regulatory complex (WRC; Chen et al., 2017). Previous studies using RNA interference in mammalian and *Drosophila* cells have indicated that all WRC subunits are essential for Rac-mediated actin remodeling (Innocenti et al., 2004; Kunda et al., 2003; Rogers et al., 2003; Steffen et al., 2004). Genetic deletion of WRC subunits in both mammalian and *Dictyostelium* cells have hitherto confirmed the key role of WRC in Rac-mediated actin remodeling (Schaks et al., 2018; Whitelaw et al., 2020), albeit with the phenotypes obtained upon targeting distinct WRC subunits in *Dictyostelium* being admittedly more heterogeneous (Bear et al., 1998; Davidson and Insall, 2014; Litschko et al., 2017). Based on all these results, the WRC has been considered crucial, if not obligatory, for Arp2/3 complex-dependent actin remodeling in cell edge protrusions that form downstream of Rac (Rottner and Schaks, 2019), although some doubts have remained. For example, *Dictyostelium* cells lacking SCAR have been reported to engage WASP instead of SCAR, although this requires increased Rac activities in these cells (Veltman et al., 2012), and *Caenorhabditis elegans* neuroblasts appear to employ both WAVE and WASP for leading edge Arp2/3 activation (Zhu et al., 2016). Furthermore, despite a lack of phenotypes concerning the efficiency of membrane ruffling and lamellipodia protrusion in different mammalian cell types lacking N-WASP (also known as WASL) or the mostly hematopoietic WASP (Leithner et al., 2016; Lommel et al., 2001; Snapper et al., 2001), mature dendritic cells display WASP accumulation at the cell periphery if forced to protrude under confinement (Leithner et al., 2016). Likewise, loss of WRC function in tumor cells migrating in 3D appears to promote N-WASP-dependent invasion (Tang et al., 2013), indicating complex, perhaps condition- and context-dependent interrelationships between the functions of the WRC and WASP and/or N-WASP in regulating Arp2/3-dependent actin remodeling at the cell periphery.

Finally, signaling to Rac-dependent actin remodeling does not always have to include formation of prominent plasma membrane protrusions, such as those occurring during membrane ruffling or lamellipodia formation. For example, the gram-positive bacterium *Listeria monocytogenes* employs a bacterial effector called Internalin B (InlB), which binds to the receptor tyrosine kinase hepatocyte growth factor receptor (HGFR, also known as c-Met or MET), stimulating Rac-dependent actin remodeling during induced bacterial host cell entry. Analogous to formation of lamellipodia, this pathway largely depends on Rac and its downstream effector WRC (Bosse et al., 2007), although a previous study could again not exclude a cell type- or condition-dependent role in entry for N-WASP (Bierne et al., 2005).

In the current study, we establish and characterize a hitherto unrecognized lamellipodia-like activity in mouse melanoma and fibroblast cell lines entirely devoid of WRC, which involves all regulators of canonical lamellipodia with the exception of Ena/VASP family actin polymerases. Our data thus establish that (1) WRC is not entirely obligatory for Arp2/3 complex-dependent actin network formation at the cell periphery and (2) WRC is essential for Ena/VASP accumulation at the tips of protrusive lamellipodia.

## RESULTS

### Nap1-null clones display distinct lamellipodial phenotypes due to differential Hem1 expression

**Fig. 1.**
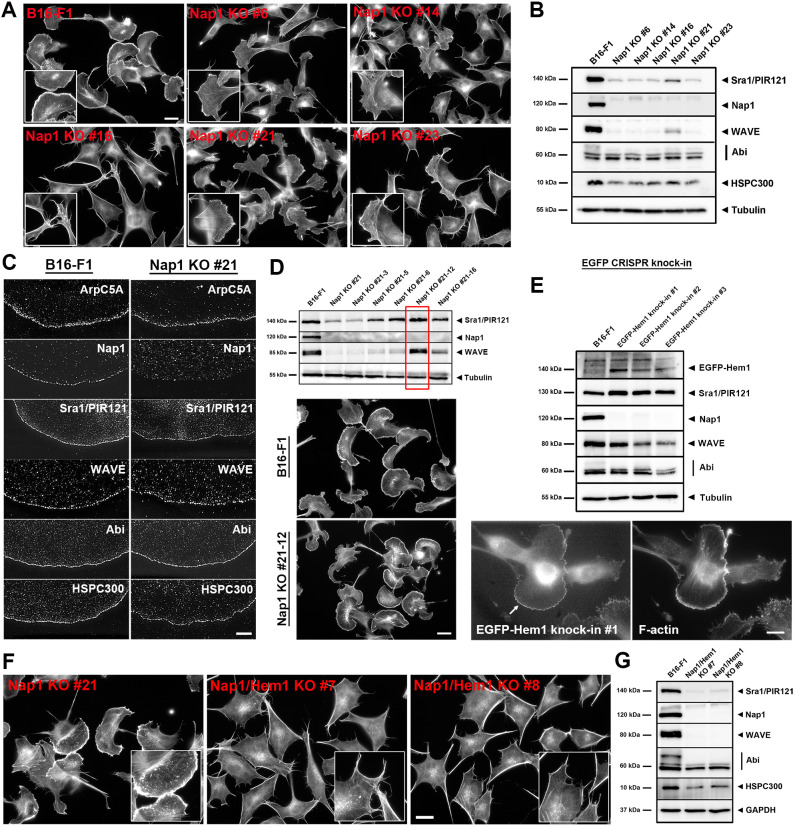
**Elimination of canonical lamellipodia in B16-F1 cells requires removal of both Nap1 and Hem1.** (A) Comparison of actin cytoskeleton morphologies of B16-F1 wild-type cells and the indicated Nap1 single KO clones stained with phalloidin. Note that Nap1 KO in B16-F1 cells altered lamellipodia frequency and morphology to variable extents in distinct clones but did not completely abrogate their formation (insets). Scale bar: 20 µm. (B) Western blotting of whole-cell extracts of Nap1 KO clones and B16-F1 cells as control to detect expression levels of other WRC subunits, as indicated (for quantitations see Fig. S1C). α-Tubulin served as loading control. (C) Representative structured illumination microscopy images of lamellipodial sections from either B16-F1 cells or Nap1 KO clone 21. Antibody staining demonstrates accumulation of Arp2/3 complex (subunit ArpC5A) and all WRC subunits besides Nap1 in lamellipodia formed by Nap1 KO clone 21. Scale bar: 5 µm. (D) Subcloning of Nap1 KO clone 21 in order to enrich for a cell population with high Hem1 expression. Despite the lack of Nap1, derived subclone 21-12 (red box, top) was found to possess expression levels of WRC subunits identical to parental B16-F1 cells, indicative of further increased compensatory Hem1 expression. α-Tubulin served as loading control. Accordingly, cellular morphologies and lamellipodia formation were indistinguishable from wild-type B16-F1 controls (bottom), as shown by phalloidin staining. Scale bar: 20 µm. (E) EGFP insertion upstream of the gene locus encoding Hem1 to give rise to a fusion protein proving robust, endogenous Hem1 protein expression in subclone 21-12 using a CRISPR-mediated knock-in approach. Top: expression of EGFP-tagged Hem1 of the expected molecular mass in three independently isolated knock-in clones, as verified by western blotting using anti-EGFP antibody alongside western blotting for other WRC subunits. α-Tubulin served as loading control. Bottom: cell images displaying the formation of lamellipodia stained with phalloidin (right) and tipped by EGFP–Hem1 (left, white arrow); representative data shown for EGFP–Hem1 CRISPR knock-in clone 1 in Nap1 KO clone 21-12 background. Scale bar: 10 µm. (F) Overview images of phalloidin-stained Nap1 KO clone 21 and two clones derived from the latter by consecutive additional CRISPR/Cas9-mediated genetic disruption of Hem1. Note that additional removal of Hem1 leads to complete loss of lamellipodia formation (insets). Scale bar: 20 µm. (G) Western blotting of whole-cell extracts of wild-type B16-F1 versus Nap1/Hem1 double KO lines, showing almost complete elimination of expression of WRC subunits Sra1/PIR121 and WAVE versus modest reduction of expression in the case of Abi1 and HSPC300 (for quantitations see Fig. S3C). Images in A,F and C,D,E are representative of three and one independent experiments, respectively. Western blots in D and E reflect results from single experiments. Western blots in B and G are representative of four and six independent experiments, respectively.

To obtain B16-F1 melanoma cell clones with abolished WRC function, as an alternative to Sra1 and PIR121 (also known as CYFIP2; referred to collectively hereafter as Sra1/PIR121) double knockout (KO) cells or WAVE protein KOs (Schaks et al., 2018; Tang et al., 2020), we initially disrupted the *Nckap1* gene encoding Nap1 (Fig. S1A), which was hitherto thought to be essential for WRC function in this cell type (Steffen et al., 2006, 2004). A subfraction of these clones (clones 6 and 21) have previously been used as tools to study cells with low lamellipodial Arp2/3 complex activity (Dolati et al., 2018) but have not been characterized in further detail. Clone 16 has also been employed recently to show that cells lacking apparent lamellipodia display a significant reduction in turnover of peripheral actin filaments (see below and Whitelaw et al., 2020), as expected (Steffen et al., 2014). Excepting clones 1 and 2, which showed strongly reduced but not abolished Nap1 expression and were thus not used further, eleven additional clones lacked apparent Nap1 expression (Fig. S1A), five of which were confirmed to lack *Nckap1* wild-type alleles by sequencing (Fig. S1B) and, thus, were analyzed further. Whereas cultures of clone 23 were virtually devoid of cells with lamellipodia, clones 16 and 14 displayed a very low percentage of cells with compromised lamellipodia, and this percentage was higher in clones 6 and 21 – almost 10% and more than 50% of cells with lamellipodia, respectively – which certainly came as a surprise (Fig. 1A; quantitation in Fig. S2; see also Dolati et al., 2018). These observations also correlated with modest but clearly detectable expression of Sra1/PIR121 and of WAVE proteins in extracts from bulk populations of clone 21 cells, which was not observed in the other clones (Fig. 1B; Fig. S1C). Expression levels of Abi proteins and HSPC300 (also known as BRK1) were also modestly reduced in these clones but were likely not limiting (Fig. 1B; Fig. S1C). Further characterization of lamellipodia formed in clone 21 cells confirmed the presence – aside from the Arp2/3 complex – of all WRC subunits except for Nap1 (Fig. 1C), suggesting that the functionality of these WRCs was achieved through expression of the mostly hematopoietic Hem1 (which is encoded by *Nckap1l*; Park et al., 2010). Indeed, despite failure to detect Hem1 in regular cell extracts of Nap1 KO clone 21, we were able to co-immunoprecipitate the protein with EGFP-tagged Sra1, just like WAVE and Abi proteins, but only in Nap1 KO clone 21 and not in wild-type B16-F1 cells (Fig. S3A). These data strongly suggest compensatory Hem1 expression in clone 21; this was even enhanced upon subcloning, giving rise to Nap1 KO clone 21-12, which displayed WAVE expression and lamellipodia formation efficiency indistinguishable from that of wild-type B16-F1 cells (Fig. 1D). To prove Hem1 expression in this clone, we CRISPR/Cas9-engineered EGFP into the gene locus encoding Hem1, and this allowed us to confirm EGFP–Hem1 expression in three independent clones by western blotting and observe its accumulation at lamellipodia tips using single-cell fluorescence microscopy (Fig. 1E).

We also employed Nap1 KO clones 16 and 23, which had very low to no lamellipodial activity and hence endogenous Hem1 expression, for comparative rescue experiments using EGFP-tagged Nap1 and Hem1 to explore the possibility of differential activities between the two proteins in regard to lamellipodia formation or cell migration. Potential differences could not be excluded, as previous studies have described a single point mutation that abrogates Hem1 function but is without effect at the corresponding sequence position in Nap1 (Salzer et al., 2020). However, the extent and frequency of lamellipodia formation (Fig. S4A,B), WRC subunit expression (Fig. S4C) as well as migration efficiency (Fig. S4D–F) were all equally efficiently restored in Nap1 KOs with either EGFP–Nap1 or EGFP–Hem1. Even the turnover of both fusion proteins at lamellipodia tips was comparably slow (t_1/2_ of ∼30 s; Fig. S4G,H).

All these data confirm that the virtual lack of lamellipodia formation in specific individual Nap1 KO clones (i.e. clones 16 and 23) is derived from absent Hem1 expression, and that differential lamellipodia formation in other clones or subclones is derived from upregulation of Hem1, which can be considered equally active as Nap1, at least concerning the lamellipodial or migration parameters analyzed here.

### Thresholds of WRC activity correlate with lamellipodia formation capability and cell migration efficiency

To obtain cells lacking lamellipodia entirely, we disrupted the locus encoding Hem1 in Nap1-null cells. For screening purposes in the absence of a highly sensitive Hem1-specific western blotting antibody, cells of Nap1 KO clone 21 were used, and derived Nap1 and Hem1 double-null cells were isolated using lamellipodial deficiency as parameter. After selection of these double KO clones (hereafter referred to as Nap1/Hem1 double KO) based on morphological parameters (Fig. 1F), two clones were subjected to sequencing of the locus encoding Hem1 (clones 7 and 8; Fig. S3B). Both clones harbored at least three alleles, all leading to gene disruption except for one in-frame deletion of residue 20 in clone 7. Due to the absence of lamellipodia in these conditions, we concluded that deletion of the aspartate residue at position 20 of Hem1 generates a non-functional variant of the protein. Expression of EGFP-tagged Hem1ΔD20 in Nap1/Hem1 double KO cells confirmed this view, since lamellipodia were not rescued by the mutant but were readily observed in the same cells transfected with EGFP-tagged wild-type Hem1 (Fig. S5). Nap1/Hem1 double KO cell lines were virtually devoid of both Sra1/PIR121 and WAVE proteins, and were reduced in expression of Abi proteins and HSPC300 to ∼50% of wild-type levels (Fig. 1G; Fig. S3C).

**Fig. 2.**
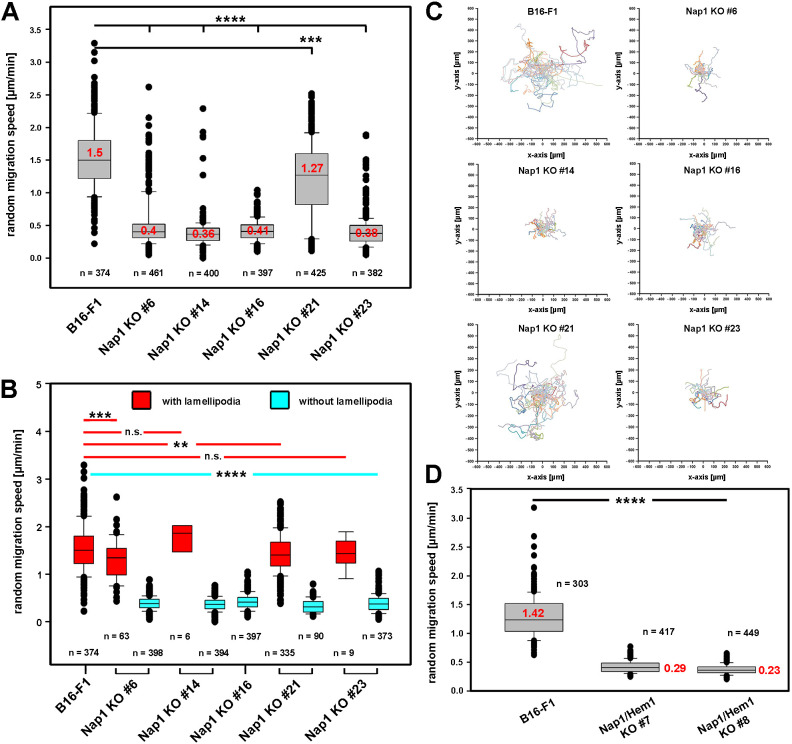
**Effective migration of B16-F1 cells closely correlates with the capability to form lamellipodia.** (A) Random migration speed of the indicated Nap1 KO clones compared to parental B16-F1 cells. Box and whisker plots with boxes corresponding to 50% of data points (25th–75th percentiles) and whiskers corresponding to 80% of data points (10th–90th percentiles). Outliers are shown as dots, and red numbers in boxes correspond to medians, which are also marked by horizontal lines. *n* cells for each clone is indicated in the figure. All Nap1 single KO clones were markedly reduced in migration, except for KO clone 21, which was only moderately affected. Yet, all changes were found to be statistically significantly different. (B) Random migration data discriminating Nap1 KO cells with (red boxes) and without (blue boxes) lamellipodia show that lamellipodia-forming Nap1 KO cells migrate at rates indistinguishable from controls, whereas lamellipodia-deficient cells display an average migration speed of 0.4 µm/min. This level was highly comparable to the migration speed of lamellipodia-deficient cells lacking Sra1/PIR121 (Schaks et al., 2018). Box plots are as described in A, with medians indicated by horizontal lines only. (C) Trajectory plots for the indicated cell lines illustrating that the frequency of lamellipodia formation revealed in B correlates with overall migration efficiency. Plots show trajectories for *n*≥20 cells per clone. (D) Random migration speed of B16-F1 cells and the indicated Nap1/Hem1 double KO clones. Note that disruption of both genes decreases migration speed even further to 0.26 µm/min on average. These data, together with previous observations on Sra1/PIR121 KO suggest additional, lamellipodia-independent functions of the Nap1–Hem1 module in migration. Box plots are as described in A. ****P≤0.0001; ***P≤0.001; **P≤0.01; n.s., *P*≥0.05 (one-way ANOVA with Dunnett’s adjustment for multiple comparisons).

Interestingly, careful quantitative analysis of random cell migration in the five Nap1 KO clones selected revealed a roughly comparable reduction of average migration speeds among clones to ∼0.4 µm/min (Fig. 2A), which was highly similar to previous observations with Sra1/PIR121 double KO cells, except for Nap1 KO clone 21 (Schaks et al., 2018). However, categorization of individually analyzed cells into those with or without lamellipodia revealed that the two subsets of cells roughly comprised two groups with astonishingly comparable migration speeds: ∼1.5 µm/min for cells with lamellipodia and roughly a third of that speed for cells without lamellipodia (Fig. 2B). The difference in clone 21 as compared to the remaining Nap1 KO clones arose from the fact that the majority of clone 21 cells displayed lamellipodia and migrated at near wild-type speed (see also Dolati et al., 2018), whereas the ratios of rapidly versus slowly migrating cells was reversed in all other clones. Clone 16 constituted the extreme case in this experiment, with no single lamellipodia-forming cell found out of 397 cells. The data thus show that the differences between clones are mostly reflected by the proportion of cells able to form lamellipodia in these migration conditions. Even in clones displaying very low overall migration rates, as illustrated in migration trajectory plots (Fig. 2C), such as for instance clone 23, those few cells capable of lamellipodia formation (*n*=9 out of 382 clone 23 cells analyzed) migrated at speeds surprisingly close to that of the majority of cells migrating with lamellipodia in the wild-type B16-F1 population (Fig. 2B). Therefore, at the individual cell level, the capability to form a canonical WRC-dependent lamellipodium was decisive for effective migration, irrespective of clone origin, and likely dependent on reaching a given threshold sufficient to form a lamellipodium (Fig. 2A–C). In Nap1/Hem1 double KO clones then, migration was not entirely abolished, but average rates were suppressed to even below 0.3 µm/min, which is consistent with the complete absence of canonical lamellipodia in all cells examined (*n*>415; Fig. 2D and data not shown).

### Growth factor stimulation can induce the formation of lamellipodia-like structures that are devoid of WRC but dependent on Rac

**Fig. 3.**
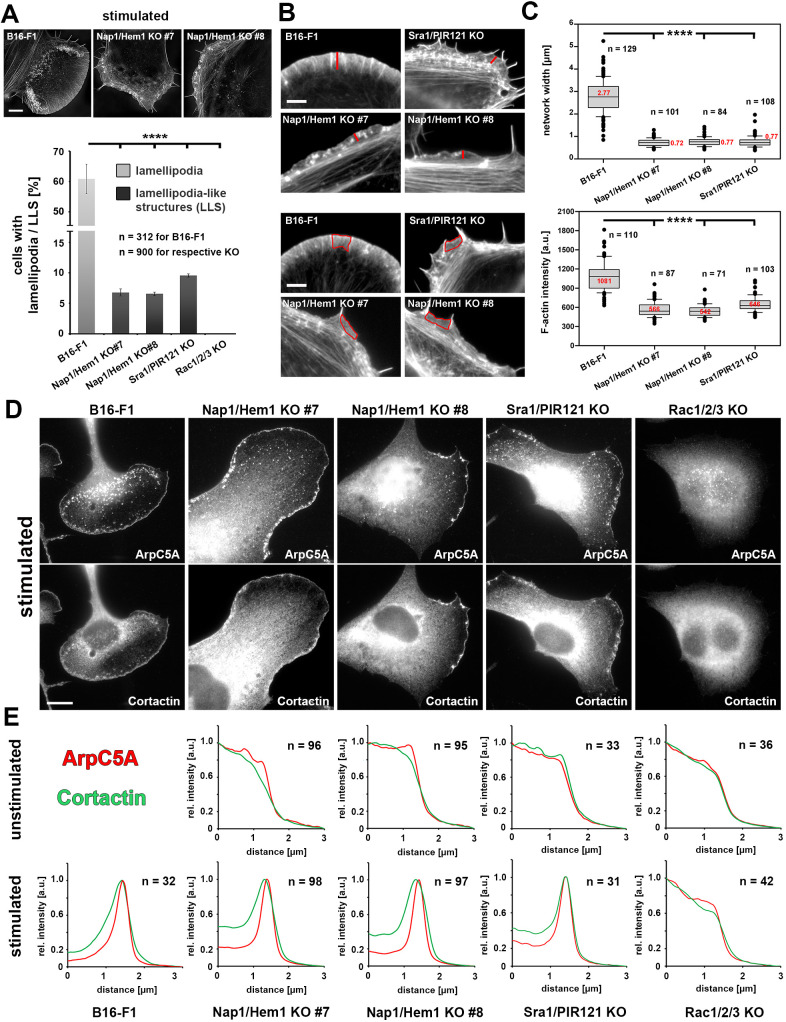
**Cells lacking WRC can form LLS upon growth factor stimulation.** (A) Top: representative structured illumination microscopy images for each of the indicated cell lines. Cells were stimulated with HGF in full growth medium, fixed and subjected to phalloidin staining. B16-F1 wild-type cells form characteristically broad, actin-dense lamellipodia with increased dorsal ruffle formation likely effected by stimulation. Scale bar: 10 µm. Bottom: bar graph showing the percentage of cells harboring either canonical lamellipodia (light gray) or LLS (dark gray) for the indicated cell lines. Note that low percentages (∼7–10%) of WRC-depleted cells were capable of forming LLS, whereas Rac1/2/3 KO cells did not form any structures reminiscent of lamellipodia. Data are arithmetic mean±s.e.m. from three independent experiments. (B) Sections of phalloidin-stained lamellipodia, in the case of B16-F1 cells, or LLS, in the case of WRC KO lines, that illustrate measurements of network width (red line; upper panel) or F-actin density (red outline; lower panel). Scale bars: 3 µm. (C) Box and whisker plots compiling the results of the measurements illustrated in B. Note that network width (upper panel) as well as F-actin density (lower panel; a.u., arbitrary units) are significantly decreased in LLS compared to canonical lamellipodia. Box and whisker plots show boxes corresponding to 50% of data points (25th–75th percentiles) and whiskers corresponding to 80% of data points (10th–90th percentiles). Outliers are shown as dots, and red numbers in boxes correspond to medians, which are also marked by horizontal lines. *n* cells for each cell line is indicated in the figure. (D) Stimulated cells with genotypes as indicated stained for endogenous Arp2/3 complex (ArpC5A) and its interactor cortactin. Note prominent accumulation of these proteins in lamellipodia of B16-F1 cells and LLS of WRC-depleted cells, but not in the absence of all three Rac subfamily members (Rac1/2/3 KO cells). Scale bar: 10 µm. (E) Graphs show the results from relative line intensity scans (a.u., arbitrary units) averaged from *n* cells, as indicated, for the experiment shown in D. ArpC5A signal intensity (red) and cortactin signal intensity (green) were measured in cells that were either stimulated or unstimulated. Measured intensity values were standardized with maximal intensities being normalized to 1 and background signals normalized to 0 for each antibody staining and individual line scan (see Materials and Methods). ****P≤0.0001 (one-way ANOVA with Dunnett's adjustment for multiple comparisons).

For more than two decades, B16-F1 melanoma cells have been regarded as a well-established model system of actin-based protrusion, migrating by employing broad and flat lamellipodia in regular growth medium when seeded on laminin (Ballestrem et al., 1998; Rottner et al., 1999a; Svitkina et al., 2003). In these conditions, we have never seen any signs of lamellipodial networks formed at the periphery of cells lacking WRC subunits (for example, see Fig. 1F and Salzer et al., 2020; Schaks et al., 2018). However, during our efforts to challenge this model with various conditions that stimulate actin remodeling, we found, to our surprise, that overnight starvation of Nap1/Hem1 double KO clones followed by treatment with growth factors in full medium triggered the formation of lamellipodia-like structures (LLS). Complementation of the growth medium with HGF, PDGF and EGF all gave comparable results, but we restricted ourselves to showing the data obtained with HGF only. The resulting structures appeared reminiscent of canonical lamellipodia, albeit less pronounced as compared to those seen in B16-F1 wild-type cells (Fig. 3A). Quantitation revealed that the frequency of LLS formation was in the range of 5–10% in these conditions, and that LLS formation was independent of how WRC was inactivated, since both Nap1/Hem1 double KO cells and Sra1/PIR121 KO cells were able to form LLS (Fig. 3A, bottom). This was in stark contrast to the frequency of canonical lamellipodia seen in wild-type cells (∼60% of cells in this experiment) or to cells lacking Rac1, Rac2 and Rac3 GTPases (Rac1/2/3 KO), in which LLS formation was abolished (Fig. 3A). Quantitation of the widths and F-actin intensities of LLS established that distinct WRC KO clones behaved highly similarly to each other with regard to these parameters, whereas lamellipodia of wild-type cells were significantly broader and contained more F-actin (Fig. 3B,C). Yet, both lamellipodia in wild-type cells and LLS found in WRC-deficient cells readily stained for the Arp2/3 complex and cortactin (also known as CTTN) (Fig. 3D), which was confirmed by line scan analyses of the cell periphery to only occur in the stimulation conditions described above (Fig. 3E). Rac1/2/3 KO cells again failed to display any signs of Arp2/3 complex or cortactin accumulation at the cell periphery (Fig. 3D,E), which is consistent with the absence of LLS formation found using phalloidin staining upon removal of Rac1, Rac2 and Rac3 (Fig. 3A, bottom panel).

**Fig. 4.**
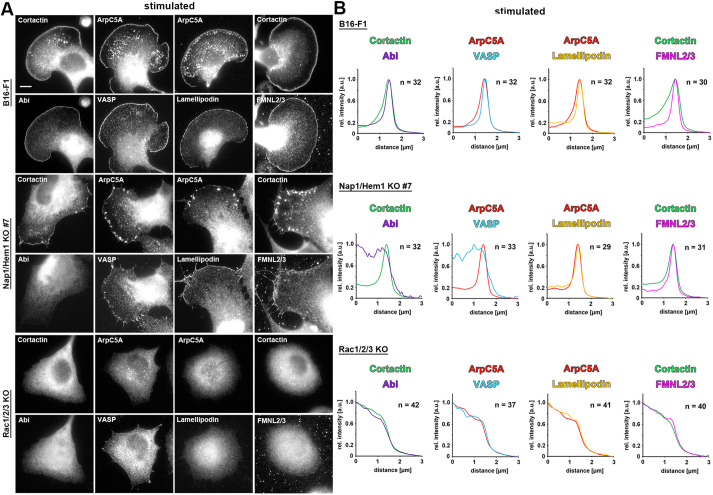
**WRC-independent LLS contain all typical lamellipodial tip components except for VASP.** (A) Representative cells with genotypes as indicated on the left were stained for lamellipodial tip proteins, including Abi, VASP, lamellipodin, and FMNL2 and FMNL3 (FMNL2/3). Depending on the species used to raise each antibody, either cortactin (detected with a polyclonal antibody) or ArpC5A (detected with a monoclonal antibody) was used as positive marker to identify lamellipodia and LLS. Scale bar: 10 µm. (B) Line scan fluorescence intensity measurements were performed for the cell stainings in A using the method described for Fig. 3E. Note the absence of specific VASP accumulation at the tips of LLS in Nap1/Hem1 double KO clone 7, which contrasts with the enrichment of VASP in a prominent line at lamellipodia tips of wild-type B16-F1 cells. None of the lamellipodial factors accumulated at the cell peripheries of Rac-deficient cells, showing that LLS are WRC-independent, but Rac-dependent. Graphs show relative line intensity scans averaged from *n* cells, as indicated (a.u., arbitrary units).

Next, we explored the accumulation of additional key lamellipodial regulators in these LLS, counterstaining as control either the Arp2/3 complex or cortactin using mono- or poly-clonal antibodies, respectively. The WRC subunit Abi (Abi1 or Abi2), the Ena/VASP family member VASP, its prominent interactor lamellipodin (Lpd, also known as RAPH1), as well as lamellipodial formins of the FMNL subfamily all readily localized at lamellipodia tips of B16-F1 wild-type cells in these conditions, as expected (Fig. 4A,B, top panels). In contrast, Abi was confirmed to be completely absent from cortactin-stained LLS in Nap1/Hem1 double KO clone 7, as expected for cells lacking a functional WRC (Fig. 4A,B), although Abi expression levels were decreased to only about half of the levels in control cells (Fig. S3C). Identical results were obtained in the two other WRC-deficient clones analyzed in this study (Fig. S6). This suggests that WRC subunits cannot be recruited to the lamellipodium in the absence of the Rac-interacting Sra1/PIR121 module or its tight interactors Nap1 and Hem1, the removal of which also mostly eliminated Sra1/PIR121 expression (Fig. S3C). Interestingly, other canonical lamellipodial components, including Lpd (Dimchev et al., 2020; Law et al., 2013) and members of the FMNL formin family – which are thought to operate independently of the Arp2/3 complex machine (Kage et al., 2017) – displayed no detectable defects of accumulation at sites of LLS formation (Fig. 4; Fig. S6). There was only one exception, the prominent Ena/VASP family of actin polymerases (Fig. 4; Fig. S6). Unlike Lpd and FMNL formins, VASP failed to accumulate in a continuous line along LLS edges (Fig. 4; Fig. S6), although it maintained the capability to target focal and nascent adhesions, which were observed to frequently align like a rope of pearls along leading edges, as previously described for vinculin-containing focal complexes (Rottner et al., 1999b). Thus, both the frequency of LLS formation and the morphological features of LLS may well derive from a combination of effects caused by removal of both WRC-mediated Arp2/3 complex activation and Ena/VASP-dependent actin assembly.

### Active Cdc42 can trigger lamellipodial structures in the absence of WRC

Previous studies have clearly shown contributions of the Rac-related GTPase Cdc42 to protrusion efficiency and actin assembly in lamellipodia, for instance through the activity of FMNL subfamily formins (Kage et al., 2017). The latter pathway, however, operates independently of Arp2/3 complex-mediated actin filament assembly and branching. In a more recent study, we have demonstrated Cdc42 to also be capable of regulating the WRC at the cell periphery in the absence of Rac, at least at low efficiency, but only when those WRCs were already rendered active due to, for example, mutation (Schaks et al., 2019a). Importantly, however, we found that expression of constitutively active Cdc42 did not stimulate detectable lamellipodia formation in the absence of Rac GTPases, in both fibroblasts (Steffen et al., 2013) and B16-F1 melanoma cells (Schaks et al., 2019a).

**Fig. 5.**
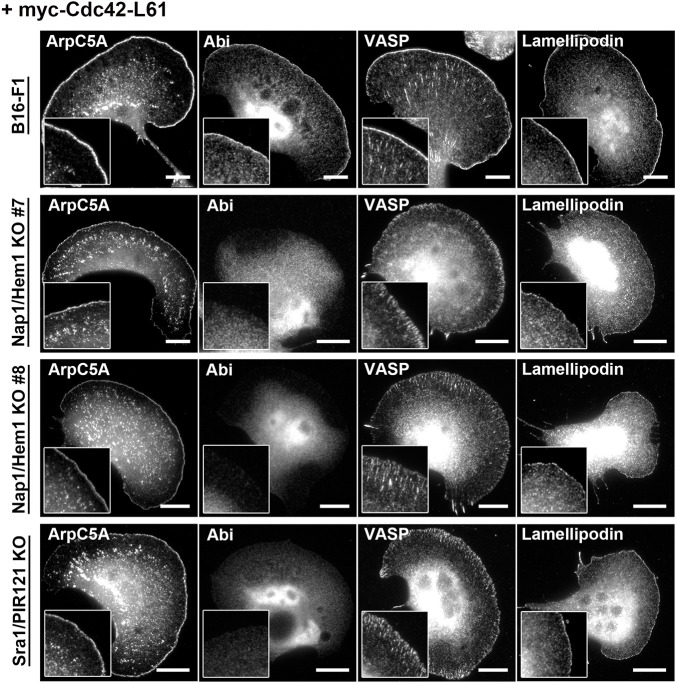
**Active Cdc42 can stimulate LLS formation in the absence of WRC.** Representative examples of B16-F1 wild-type cells and different WRC-depleted cell lines transfected with constitutively active myc–Cdc42-L61. Expression of the latter was verified by anti-myc staining (data not shown). Note that expression of Cdc42-L61 drives formation of LLS in all WRC-null cell lines without growth factor stimulation. However, like growth factor-induced LLS, these protrusions are positive for accumulation of canonical markers, such as Arp2/3 complex (ArpC5A) and lamellipodin (insets), but lack the tip enrichment of Abi and VASP seen in wild-type cells (insets). In contrast, VASP localization to nascent and mature focal adhesions is not abrogated by WRC depletion.Images are representative of two or three experiments for B16-F1 and Sra1/PI121 or Nap1/Hem1 double KO clones, respectively. Scale bars: 10 µm.

However, when expressing constitutively active Cdc42-L61 in the WRC-deficient clones described above, we found a fraction of cells (for quantitation see below) that clearly displayed polarized cell morphologies with LLS, the molecular composition of which appeared identical to the LLS seen upon serum and growth factor stimulation (Fig. 5). Aside from actin, these lamellipodial networks again included accumulation of Arp2/3 complex and Lpd within or along their edges, respectively, but lacked the WRC subunit Abi. As opposed to Lpd, which was enriched at the very edge of the lamellipodial networks, VASP was present only close to the cell periphery and was restricted to numerous nascent, arrow- or dot-shaped adhesions (Fig. 5), but again was lacking as a continuous line from the lamellipodium tip, as is commonly observed in wild-type cells (Rottner et al., 1999b; Svitkina et al., 2003).

### Cdc42-driven lamellipodial structures require the direct Cdc42 effector N-WASP

**Fig. 6.**
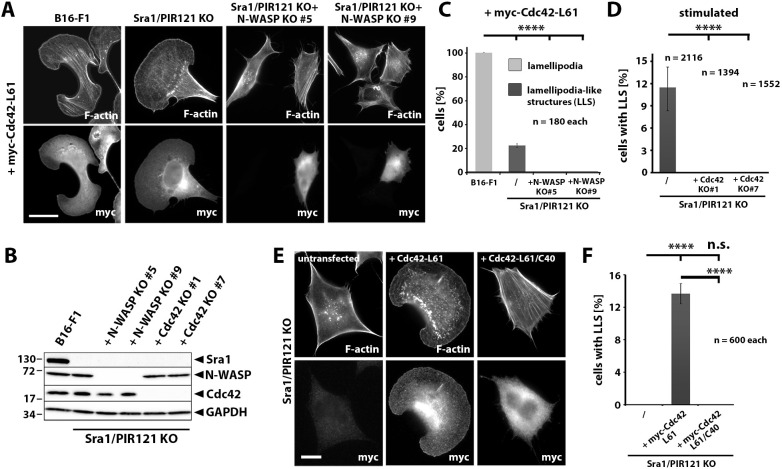
**Cdc42-stimulated LLS formation requires N-WASP, and growth factor-triggered LLS formation requires Cdc42.** (A) Expression of constitutively active Cdc42 (myc–Cdc42-L61) drives the formation of lamellipodia in wild-type B16-F1 cells and LLS in WRC-deficient cells (Sra1/PIR121 KO), but not upon additional removal of N-WASP (+N-WASP KO). Cells transfected with myc-tagged, constitutively active Cdc42 (bottom panel) were counterstained for the actin cytoskeleton using fluorescently labeled phalloidin (upper panel). Scale bar: 20 µm. (B) Western blot of CRISPR/Cas9-modified cell lines employed in the experiments shown in this figure. Removal of Sra1/PIR121 (KO clone 3; Schaks et al., 2018) was complemented by disruption of N-WASP or Cdc42, as indicated. GAPDH was used as loading control. Molecular mass is indicated in kDa. Following multiple confirmations of individual gene knockouts, compilation shown corresponds to a single, representative experiment. (C) Quantitation of the percentage of cells with lamellipodial or LLS phenotypes for the experiment shown in A. Note that 100% of wild-type B16-F1 cells transfected with constitutively active Cdc42 display a lamellipodial phenotype, which is strongly reduced (but not abolished) to ∼20% of cells with an LLS phenotype in the absence of WRC and is completely abolished in clones additionally lacking N-WASP. Data are arithmetic mean±s.e.m. from three independent experiments, with *n*=180 cells per cell line in total. (D) Quantitation of LLS formation in WRC-deficient cells (Sra1/PIR121 KO) upon stimulation (full medium with HGF), revealing that LLS formation, occurring at a frequency of <12% under these conditions, is eliminated upon Cdc42 removal (two independent clones). Data are arithmetic mean±s.e.m. from three independent experiments, and the total number of cells analyzed for each cell line is indicated on the graph. (E) Sra1/PIR121 KO cells, either untransfected or transfected with different myc-tagged Cdc42 mutants, as indicated, were stained to detect the myc tag and F-actin (using fluorescently labeled phalloidin). Constitutively active Cdc42-L61 loses the ability to induce LLS formation in these cells when mutated at a major residue (C40) required for CRIB domain (as present in N-WASP) binding. Scale bar: 10 µm (F) Quantitation of the data displayed as representative images in E. Bar chart shows the percentages of cells harboring LLS. Data are arithmetic mean±s.e.m. from three independent experiments, with *n*=600 cells per cell line in total. ****P≤0.0001; n.s., *P*≥0.05 (one-way ANOVA with Dunnett's adjustment for multiple comparisons).

Our results prompted us to dissect the signaling to Arp2/3 complex-dependent actin assembly in WRC-deficient cells more carefully. Previously published data indicate that stimulation with growth factors such as HGF, as used above, can elicit signaling to Rho GTPase-dependent actin remodeling, such as lamellipodia formation and membrane ruffling, and this includes activation of the prominent Rho family GTPase Cdc42 (Bosse et al., 2007; Royal et al., 2000). To this end, we disrupted either Cdc42 or the Cdc42 effector N-WASP in a B16-F1 clone already lacking functional WRC through Sra1/PIR121 KO (clone 3; Fig. 6A,B) (Schaks et al., 2018). We examined two independently generated clones for each novel genotype in more detail (Fig. 6B). Interestingly, side-by-side comparison of cells transfected with myc-tagged, constitutively active Cdc42 revealed that B16-F1 wild-type cells and Sra1/PIR121 KO clone 3 displayed similar morphologies of cells positive for the myc tag (Fig. 6A). However, quantification revealed that the percentage of cells displaying this phenotype dropped from 100% in wild-type cells to ∼20% in cells lacking WRC (Fig. 6C). This suggested that the majority of lamellipodia formed in B16-F1 wild-type cells and additionally boosted by active Cdc42 were likely formed through WRC and concomitant Rac activation, as expected from previous work (Nobes and Hall, 1995; Rottner and Stradal, 2011), but there were clear exceptions not requiring WRC (Fig. 6A,C). Those exceptions formed in Sra1/PIR121-null cells were entirely eliminated upon additional removal of the Cdc42 effector and prominent Arp2/3 complex activator N-WASP (Rohatgi et al., 1999) (Fig. 6A,C). Finally, stimulation of starved cells with the serum and growth factor cocktail used in previous experiments (Figs 3, 4; Fig. S6) revealed that the ∼10% of Sra1/PIR121-deficient cells displaying LLS in this experiment were entirely eliminated by additional genetic removal of Cdc42 (Fig. 6D). Likewise, when replacing the tyrosine residue at position 40 of Cdc42 with cysteine (Cdc42-L61/C40), which is known to abolish interaction with any Cdc42- and Rac-interactive binding (CRIB) domain-containing effector, such as N-WASP, LLS formation in Sra1/PIR121 KO cells was abolished (Fig. 6E,F). Taken together, these observations lead us to conclude that LLS thus constitute peculiar Arp2/3 complex-containing actin networks formed at the cell periphery that essentially require both Rac and Cdc42 signaling, and the formation of which is normally superimposed by WRC activity in wild-type B16-F1 cells. Importantly, these structures can contribute to the efficiency of cell migration, since WRC-deficient cells additionally disrupted for Cdc42 displayed a statistically significant reduction in random cell migration speeds (Fig. S7A), which was not seen in B16-F1 clones solely lacking Cdc42 (Fig. S7B,C).

### LLS stimulation and Rac-dependent, but WRC-independent, actin remodeling activities are recapitulated in fibroblasts

In order to examine whether compensatory expression of Hem1 upon Nap1 deletion, as well as the peculiar capability of LLS formation in Nap1/Hem1 double KO cells, was restricted to B16-F1 melanoma cells, we chose the commonly employed NIH 3T3 fibroblast cell line as independent cell type. Although mouse embryonic fibroblasts (MEFs) have been described previously as virtually devoid of lamellipodia upon acute, tamoxifen-mediated deletion of the *Nckap1* gene encoding Nap1 (Whitelaw et al., 2020), isolated NIH 3T3 clones identified by western blotting as lacking Nap1 expression following CRISPR/Cas9 treatment (Fig. S8A; clones 1, 5 and 8) still invariably displayed tiny, but clearly detectable, lamellipodia (Fig. S8B). Next, we disrupted Hem1, starting from the NIH 3T3 Nap1 KO clone 8, and identified two consecutive Nap1/Hem1 double KO clones (2 and 3) that were clearly devoid of any detectable lamellipodia in regular growth conditions (Fig. S8C). Sequencing of the locus encoding Hem1 confirmed the absence of any wild-type allele (Fig. S8D). Moreover, western blotting of all five WRC subunits revealed both a clear but somewhat differential reduction of remaining subunits in the Nap1 single KO clone 8, and even further reduction – if not elimination, as observed for WAVE proteins – of remaining subunits in both double KO lines (Nap1/Hem1 double KO clones 2 and 3; Fig. S8E). Consistent with formation frequencies of canonical lamellipodia (Fig. S8B,C) and in analogy to the B16-F1 data above (Fig. 2D), we found a significant inhibition of random cell migration speed in these fibroblasts, down to ∼25% of the control levels in the two Nap1/Hem1 double KO clones, and an intermediate phenotype in the precursor Nap1 KO clone 8 (Fig. S8F).

**Fig. 7.**
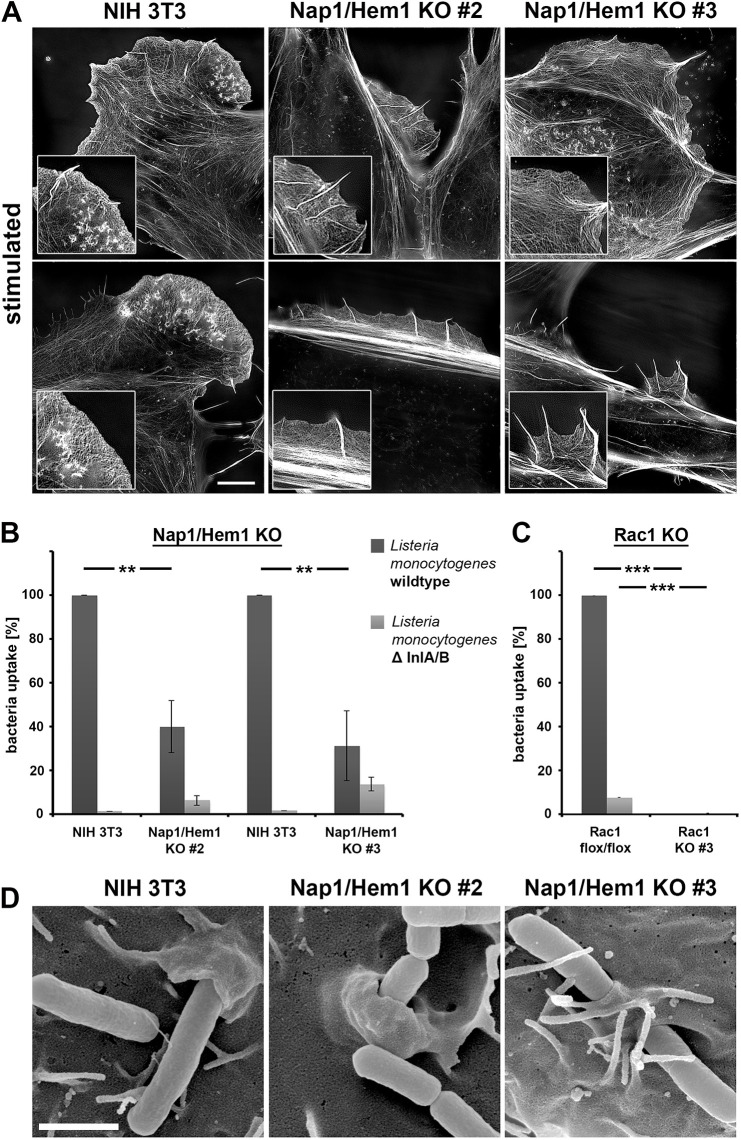
**Nap1/Hem1 double KO fibroblasts can form LLS and are partially susceptible to host cell invasion by *L. monocytogenes*.** (A) Structured illumination microscopy images of stimulated cells (HGF in full medium) stained for the actin cytoskeleton with fluorescently labeled phalloidin (two representative images per cell line). Note that both Nap1/Hem1 double KO clones, although lacking functional WRC, can form peripheral structures reminiscent in appearance and dimension of the lamellipodia in wild-type NIH 3T3 cells (left), demonstrating that this phenomenon is not restricted to B16-F1 cells. Insets show lamellipodia for wild-type NIH 3T3 and LLS for Nap1/Hem1 double KOs. Images are representative of three experiments. Scale bar: 5 μm. (B) NIH 3T3 control cells and the indicated KO fibroblasts were tested for invasion efficiency of wild-type *L. monocytogenes* versus InternalinA/B-deficient bacteria (ΔInlA/B) as control, using gentamicin survival assays. Data are arithmetic mean±s.e.m. from four independent experiments, each normalized to the uptake of wild-type bacteria by the parental NIH 3T3 cells. (C) *L. monocytogenes* invasion assay as in B but performed on Rac1^flox/flox^ control MEFs and on Rac1 KO clone 3 MEFs lacking Rac activity (Steffen et al., 2013). Data are arithmetic mean±s.e.m. from three independent experiments, normalized to uptake of wild-type *L. monocytogenes* by Rac1^flox/flox^ cells. Note that loss of WRC reduces *L. monocytogenes* invasion to ∼30–40% (as shown in B), whereas loss of Rac (Rac1 KO clone 3) abolishes both specific (wild-type *L. monocytogenes*) and non-specific (ΔInlA/B) pathogen uptake. In B and C, **P≤0.01; ***P≤0.001 (Mann–Whitney rank sum test). (D) Representative scanning electron microscopy images showing *L. monocytogenes* (cylinder-like objects) uptake by the indicated cell lines. Despite the reduced uptake efficiency upon Nap1/Hem1 double KO as compared to control (see B), little difference in the morphology of cell surface structures accompanying bacterial entry could be observed. The variability in the formation frequency of microvilli-like structures in distinct clones was independent of genotype (compare Nap1/Hem1 double KO clones 2 and 3). Images are representative of ∼20 observed entry events per cell line from two independent experiments each. Scale bar: 1 µm.

Most strikingly, and as opposed to the phenotypes observed in regular growth conditions (Fig. S8C), starvation followed by serum plus growth factor stimulation (again HGF in this case) resulted in the formation of reasonably prominent LLS containing microspike bundles in both Nap1/Hem1 double KO clones (see insets in Fig. 7A). Assuming that the molecular regulation of these structures would be consistent with our observations described above for B16-F1 melanoma, we sought instead to explore the physiological relevance of LLS formation in NIH 3T3 fibroblasts in a complementary model system of active actin remodeling at the plasma membrane.

*L. monocytogenes* invades host cells using bacterial surface or secreted proteins known as internalins. Due to the lack of interaction between Internalin A (InlA) and the murine version of its host cell ligand, E-cadherin, invasion of murine cells is exclusively mediated through InlB, which elicits Rac-dependent actin remodeling essential for entry downstream of c-Met (Pizarro-Cerda et al., 2012). Based on a fibroblast cell line stably suppressed for Nap1 expression by RNA interference, we previously concluded that InlB-triggered actin remodeling requires WRC and Nap1 (Bosse et al., 2007). However, here we clearly demonstrate, by employing our newly generated Nap1/Hem1 double KO cell lines, that loss of functional WRC (Fig. S8E) reduces, but does not entirely eliminate, the InlB–c-Met pathway of bacterial invasion, as compared to invasion of control NIH 3T3 cells (Fig. 7B). The difference between wild-type *L. monocytogenes* and isogenic mutants lacking Internalin A and B (ΔInlA/B) proved the specificity of the response for InlB–c-Met signaling (Fig. 7B). As seen with growth factor-induced LLS formation in B16-F1 cells, this type of actin remodeling was again completely eliminated in cells lacking Rac GTPases (Steffen et al., 2013), confirming the pattern of Rac-dependent but WRC-independent actin remodeling events uncovered in the context of the current experiments (Fig. 7C). Finally, since the efficiency of bacterial entry was reduced to ∼30–40% in the absence of WRC, we asked whether those entry events occurring in the absence of WRC were by any means morphologically distinguishable from those in control fibroblasts. However, scanning electron micrographs capturing bacteria right in the middle of the entry process revealed that, except for some variability in formation of microvilli-like structures routinely seen in these fibroblast cells and partly accompanying invasion, no major alterations were seen in the size and shape of the plasma membrane leaflets wrapping around invading bacteria (Fig. 7D). These data are consistent at least with the concept of *L. monocytogenes* invasion being mediated by a so-called zipper mechanism of entry (Mostowy and Cossart, 2009), with invading bacteria sinking into their host cells, with plasma membrane leaflets zippering around them. It is tempting to speculate that despite essential Rac functions, the potency and efficiency of WRC-mediated Arp2/3 complex activation and actin assembly is not obligatory for these spatially confined actin remodeling events, which is in agreement with previously published conclusions (Bierne et al., 2005).

## DISCUSSION

For nearly two decades now, WRC-dependent canonical lamellipodia have been considered the key structures mediating effective cell motility, in particular that occurring downstream of Rac GTPase signaling. This is true for an overwhelming majority of studies focusing on canonical migration *in vitro* and in 2D, whereas a seminal study on epithelial tumor cell migration in 3D has already uncovered mutual antagonisms between classical, WRC–Arp2/3-dependent migration and N-WASP–Arp2/3-dependent tissue invasion (Tang et al., 2013). Our studies using CRISPR/Cas9-mediated gene editing in various combinations allowed us to demonstrate here that WRC can indeed be bypassed to a certain extent for Arp2/3 complex-dependent actin assembly at the cell periphery even *in vitro* and in 2D, under particular circumstances. This occurs, for instance, in cells lacking functional WRC that are subjected to growth factor stimulations, such as with HGF, or to transfection with constitutively active Cdc42. In such and similar experimental conditions, we were able to observe the formation of lamellipodia-like actin networks in the absence of WRC; admittedly this occurred at comparatively low frequency, but this phenomenon has hitherto not been seen at all in any mammalian model cell line protruding and migrating in 2D. These observations thus challenge the current paradigm of the well-established, 1D signaling axis to lamellipodia formation requiring Rac, WRC and the Arp2/3 complex (Schaks et al., 2019b; Steffen et al., 2014).

**Fig. 8.**
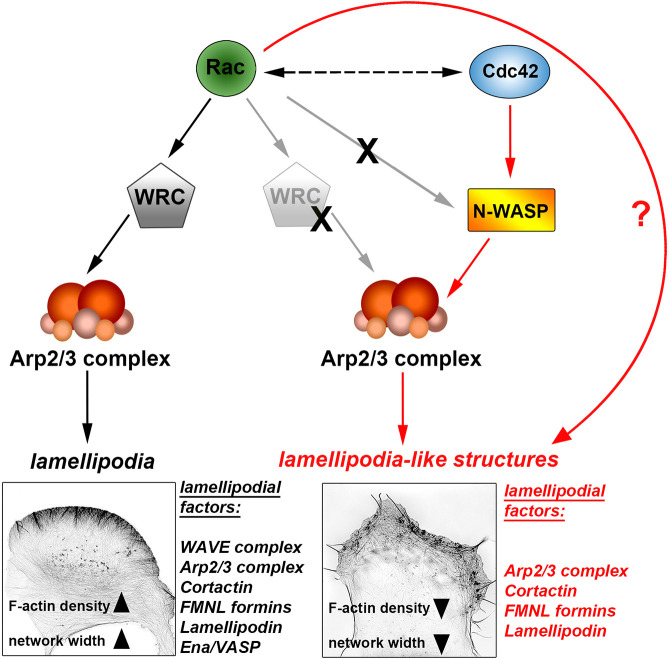
**Graphical summary of pathways driving lamellipodia formation versus LLS formation.** The canonical pathway leading to lamellipodia formation (black arrows) in cells involves active Rac GTPase (green) signaling to recruit and activate the pentameric WRC, which in turn activates the heptameric Arp2/3 complex, the major actin nucleator in lamellipodia (black arrows). In the absence of WRC, cells cannot activate Arp2/3 complex by canonical, WRC-mediated activation (crossed-out WRC), yet are able to form LLS, at least in specific conditions (for example upon activation of Cdc42, blue), challenging the classical paradigm of lamellipodia formation. LLS formation (red arrows) also involves Arp2/3 complex, which downstream of Cdc42 is activated by N-WASP but not Rac (crossed-out gray arrow). However, despite Cdc42 operating through N-WASP, LLS formation still requires Rac activity in a pathway that remains to be established (red line and question mark). Aside from this, Rac and Cdc42 GTPases may also undergo significant crosstalk (black dashed line), which also requires further specification. Note that the pathway leading to LLS formation is less efficient than the classical, WRC-driven pathway, and this manifests as (1) lower overall frequency of formation, (2) reduced F-actin density and (3) reduced actin network width (see representative images and Table S1). This may be due, at least in part, to the absence of the Ena/VASP family of actin polymerases in LLS, which is prominent, however, in lamellipodia (left). Aside from WRC and Ena/VASP, LLS harbor all canonical lamellipodial factors tested (red list on the bottom right). Representative images show F-actin staining (using fluorescent phalloidin) for a wild-type B16-F1 cell on the left, and a Nap1/Hem1 double KO cell on the right.

Importantly, however, these WRC-independent LLS still required as an essential prerequisite the presence of any of the three mammalian Rac GTPases and, in addition, the related GTPase Cdc42 (see model in Fig. 8). One apparent distinction between LLS formation and lamellipodia formation is that Rac alone can trigger the formation of canonical lamellipodia through WRC activation and Arp2/3 complex-dependent actin assembly, which is essential for efficient cell migration in the model systems employed here (black arrows in Fig. 8). In the absence of WRC, however, both Rac and Cdc42 are obligatory for the formation of LLS (red arrows), which are smaller in dimension and formed less frequently than canonical, WRC-dependent lamellipodia (see summary of all results and quantifications in Table S1). Despite the essential function of Rac, it is Cdc42 that has to signal through N-WASP in Cdc42-triggered LLS formation, whereas the precise target and role of Rac in LLS formation remains to be elucidated. In wild-type cells, active Cdc42 expression can raise the proportion of cells displaying a lamellipodial phenotype to 100%, but this number is strongly inhibited down to ∼20% upon WRC removal (for example, see Fig. 6C). The relevance of Cdc42 for actin remodeling at the cell periphery, in particular in the absence of canonical lamellipodia, is emphasized by its gain of impact on cell migration in the absence of WRC (Fig. S7).

Importantly, our observations are not restricted to migrating melanoma cells, but are mirrored in fibroblasts probed for processes requiring dynamic actin remodeling at the plasma membrane, including responses induced by growth factor stimulation or bacterial invasion. It is well established that InlB-mediated *Listeria* invasion requires Rac signaling and Arp2/3 complex-dependent actin remodeling on the cell surface. Along that line, we found that *Listeria* uptake by fibroblasts lacking Rac was entirely abrogated; however, uptake was only reduced to ∼40% in WRC-deficient fibroblast cells. Given that we could trigger LLS formation by HGF treatment, and that *Listeria* invasion through InlB is known to occur through engagement of c-Met, the HGF receptor, we explored the physiological relevance of this pathway for actin remodeling events stimulated by bacterial entry. Although being less efficient than the canonical pathway, the c-Met signaling pathway could still be exploited for bacterial invasion in the absence of WRC, highlighting the physiological relevance of this unconventional bypass route to actin remodeling downstream of the HGF receptor, Cdc42 and N-WASP.

The WRC-independent lamellipodia-like structures described in this study harbor all classical, lamellipodial factors tested, including Arp2/3 complex, but with the exception of Ena/VASP family proteins. VASP was the first factor to be directly associated with promoting actin polymerization at the edge of protrusions, such as lamellipodia and filopodia, or at the surfaces of bacteria recruiting parts of the actin assembly machinery for intracellular motility (Lambrechts et al., 2008; Laurent et al., 1999; Rottner et al., 1999a; Svitkina et al., 2003). Despite resulting in changes to lamellipodial architecture and size, disruption of all three mammalian Ena/VASP family members does not abolish lamellipodia formation entirely (Damiano-Guercio et al., 2020). Previously observed phenotypes of Ena/VASP loss of function, for instance the reduction of lamellipodial actin filament mass or lamellipodial width, are certainly consistent with the observations described here for LLS lacking VASP at their tips. Interestingly, Ena/VASP proteins have indeed previously been concluded to operate downstream of WRC in *Dictyostelium* (Litschko et al., 2017). Furthermore, Ena/VASP family member recruitment downstream of WRC might occur through their direct interactions with the WRC subunit Abi, which has previously been established in various model organisms (Chen et al., 2014; Litschko et al., 2017; Shi et al., 2021). However, due to the complete abrogation of lamellipodia formation in normal growth conditions upon WRC removal (Schaks et al., 2018; Tang et al., 2020) and the dependence of lamellipodial Ena/VASP accumulation on the presence of a lamellipodium (Rottner et al., 1999a), formal proof of WRC operating upstream of VASP accumulation in protruding membranes – but not nascent adhesions – has hitherto been lacking.

With the data presented here, we formally prove (1) that WRC-mediated Arp2/3 complex activation is not obligatory for actin remodeling downstream of Rac GTPase signaling at protruding membranes, unlike current concepts in the field, and (2) that WRC can operate as signaling hub at the leading edge of lamellipodia, likely aiding in the accumulation of relevant actin assembly factors, including VASP.

## MATERIALS AND METHODS

### Cell culture and transfections

B16-F1 murine melanoma cells (ATCC CRL-6323) were cultured in DMEM (4.5 g/l glucose; Invitrogen), supplemented with 10% fetal calf serum (FCS; Gibco, Paisley, UK) and 2 mM L-glutamine (Thermo Fisher Scientific). Sra1/PIR121 KO and Rac1/2/3 KO cells were as described previously (Schaks et al., 2018). Transfections of parental B16-F1 cells were carried out in 35 mm dishes using 0.5 µg DNA and 1 µl JetPrime transfection reagent (PolyPlus), whereas 1 µg DNA and 2 µl JetPrime were required to transfect respective KO cell lines. After overnight transfection, B16-F1 cells were seeded onto glass coverslips pre-coated with laminin (Sigma, L2020; 25 µg/ml in laminin-coating buffer: 150 mM NaCl, 50 mM Tris-HCl, pH 7.4).

NIH 3T3 (ATCC CRL-1658) and derived KO cell lines as well as Rac1^fl/fl^ MEFs and the corresponding Rac1 KO (clone 3) (Steffen et al., 2013) were grown in DMEM (4.5 g/l glucose), 10% FCS (Sigma), 2 mM L-glutamine, 1% non-essential amino acids and 1 mM sodium pyruvate. NIH 3T3 cells and corresponding KO clones were transfected with a total of 3 µg DNA and 9 µl JetPrime in 35 mm dishes overnight. For microscopy, Rac1^fl/fl^ and Rac1 KO MEFs as well as NIH 3T3 cells were seeded in regular growth medium onto coverslips coated with fibronectin [Roche; 25 µg/ml in phosphate-buffered saline (PBS)]. All cell lines were cultivated at 37°C in the presence of 7.5% CO_2_. Employed cell lines were routinely authenticated and confirmed to be mycoplasma-free on a biannual basis.

### Plasmids

EGFP-C1, -C2, -C3 and EGFP-N1, -N2, -N3 vectors used for cloning were purchased from Clontech Inc. (Mountain View, CA, USA). EGFP-tagged murine Nap1 and Sra1 were described previously (Steffen et al., 2004), and EGFP–N-WASP was described in Lommel et al. (2001). EGFP–Hem1 was generated using clone IRAK961M1081Q2 obtained from the German Research Centre for Genome Research (RZPD). PRK5-myc-Cdc42-L61 was a kind gift from Laura Machesky (Beatson Institute, Glasgow, UK). This plasmid was used as template for site-directed mutagenesis of Cdc42-L61/C40 using sense oligo 5′-CTGTTTTTGACAACTGTGCAGTCACAGTTAT-3′ and respective antisense oligo. The ΔD20 mutant of EGFP–Hem1 was generated by site-directed mutagenesis using Phusion polymerase employing forward primer 5′-GCTCACGATCCTGAACCGAGGACAGGGAG-3′ and respective complementary sequence as reverse primer.

### CRISPR/Cas9-mediated genome editing

B16-F1 cells as well as NIH 3T3 cells were genome-edited using CRISPR/Cas9 technology in order to disrupt expression of Nap1 (encoded by *Nckap1*), Hem1 (*Nckap1l*), N-WASP (*Wasl*) or Cdc42 (*Cdc42*) in different B16-F1 line backgrounds. Generation of B16-F1 cells eliminated for Nap1 and Sra1/PIR121 expression has been described previously (Dolati et al., 2018; Schaks et al., 2018). Disruption of the *Nckap1* gene in NIH 3T3 cells was performed using a construct harboring the same CRISPR gRNA sequence as previously described with B16-F1 (5′-GACGCCCCGGTCGTTGAGGA-3′). In this case, isolated and expanded single-cell colonies were screened for the absence of Nap1 by western blotting using polyclonal Nap1-specific antibodies, and clones lacking detectable Nap1 expression were further verified by sequencing of the *Nckap1* locus. To obtain cell lines devoid of both Nap1 and Hem1 expression, consecutive targeting of the Hem1 gene was performed in B16-F1 Nap1 KO clone 21 (Dolati et al., 2018) and NIH 3T3 Nap1 KO clone 8 (this study) using guide sequence 5′-CTCACGATCCTGAATGACCG-3′. Due to the lack of a functional Hem1 antibody, Hem1 KO clones were initially screened for a complete loss of lamellipodia in regular growth conditions, as judged upon fixation and staining of the actin cytoskeleton with phalloidin, followed by validation of the loss of alleles giving rise to functional Hem1 expression.

Cdc42-deficient B16-F1 clones were generated using CRISPR gRNA 5′-ACAATTAAGTGTGTTGTTGT-3′. Sra1/PIR121-deficient B16-F1 clones additionally lacking Cdc42 or N-WASP were generated by targeting Sra1/PIR121 KO clone 3 (Schaks et al., 2018) with the aforementioned Cdc42 CRISPR gRNA or with CRISPR gRNA 5′-CACGTTGGTGACCCTCCGCG-3′ (for N-WASP). Screening for clones lacking Cdc42 or N-WASP expression was performed by western blotting using Cdc42-specific or N-WASP-specific polyclonal antibodies, respectively. Selected clones were validated by sequencing as described above.

Genes were routinely disrupted by targeting exon 1, and selected guide sequences were cloned into pSpCas9(BB)-2A-Puro (Addgene 48139). Following overnight transfections (described above), cells were subjected to selection pressure using puromycin (2.5 µg/ml for B16-F1 and 3 µg/ml for NIH 3T3), and then cloned by the limited dilution method (seeding density into 96 well-plates of 0.5 cells/well).

### CRISPR/Cas9-mediated EGFP knock-in

Nap1 KO subclone 21-12 was chosen for EGFP insertion into the endogenous locus encoding Hem1, as this was found to exert the highest compensatory Hem1 expression among all clones. CRISPR guide sequence 5′-TTTGGGTCTTGTTATCCGGA-3′, which is localized shortly upstream of the Hem1 start codon, was used for defined targeting of Cas9. The sequence encoding EGFP as well as ∼300 bp of arms of homology on both sides were ordered to be synthesized by Eurofins Genomics (Ebersberg, Germany). The respective CRISPR gRNA and linearized insert were co-transfected into cells at a ratio of 1:3, and cells were further processed as described in the previous section.

### Western blotting

For preparation of whole cell extracts, cells were washed thrice with ice-cold PBS, lysed using 4× SDS sample buffer (Laemmli), heated for 5 min at 95°C and sonicated to shear genomic DNA. Western blotting was carried out following standard procedures and using the following primary antibodies: anti-GAPDH (Calbiochem, clone 6C5, #CB1001; 1:10,000 dilution), anti-Cdc42 (Cell Signaling Technology, #2462; 1:1000 dilution), anti-α-tubulin (Synaptic Systems, clone 3A2; #302211; 1:5000 dilution), anti-Sra1/PIR121 (homemade rabbit polyclonal raised against peptide 4955B; 1:10,000 dilution; Steffen et al., 2004), anti-Nap1 (homemade rabbit polyclonal raised against peptide 4953B; 1:5000 dilution; Steffen et al., 2004), anti-Abi (homemade rabbit polyclonal, E3B1; 1:2000 dilution; Schaks et al., 2018), anti-pan-WAVE (home-made rabbit polyclonal; pAB5502; 1:1000 dilution; Schaks et al., 2018), anti-HSPC300 (Abcam, ab87449; 1:1000 dilution), anti-N-WASP (homemade rabbit polyclonal raised against peptide 385-401; 1:400 dilution; Lommel et al., 2001), GFP (Roche, 1814460; 1:2000 dilution) and anti-Hem1 (homemade rabbit polyclonal; kindly provided by Dr Jan Faix, Hannover Medical School, Hannover, Germany; 1:500 dilution; Stahnke et al., 2021).

HRP-conjugated secondary antibodies were anti-mouse IgG (Dianova, #115-035-062; 1:10,000 dilution) or anti-rabbit IgG (Dianova, #111-035-045; 1:10,000 dilution). Chemiluminescence signals were obtained upon incubation with ECL Prime Western Blotting Detection Reagent (GE Healthcare) and were recorded with an ECL Chemocam imager (Intas, Germany). Densitometric quantification of protein expression as detected by western blotting was essentially performed following standard procedures using MetaMorph (Molecular Devices, USA). In brief, digitalized background signals were subtracted from specific band intensities and resulting intensities subsequently normalized to those of corresponding loading controls (GAPDH or α-tubulin, as indicated). Data were plotted as bar charts using Excel 2010 (Microsoft).

### Immunoprecipitation

Commercially available GFP-TrapA (ChromoTek) was used to immunoprecipitate EGFP-tagged Sra1 or EGFP alone as control and probed for potential co-immunoprecipitation of other WRC subunits. B16-F1 cells were transfected overnight as described above. Cells were then lysed using IP buffer [140 mM KCl, 50 mM Tris-HCl (pH 7.4), 50 mM NaF, 10 mM Na_4_P_2_O_7_, 2 mM MgCl_2_] supplemented with 1% Triton-X100 and a mini cOmplete protease inhibitor pill (Roche). Lysates were centrifuged at 20,000 ***g*** for 15 min at 4°C. 5 μl of each cell lysate was mixed with SDS sample buffer, referred to as input. 30 μl bead slurry was washed 3× and spun down (2500 ***g*** for 2 min at 4°C). Subsequently, cell lysates were added to the beads and incubated under constant mixing for 1 h at 4°C. After centrifugation, 5 μl of each sample was incubated with sample buffer and used as control for unbound, EGFP-tagged proteins (supernatant). Finally, beads were washed thrice with IP buffer prior to addition of 25 μl 8× SDS sample buffer. All samples were heated for 5 min at 95°C prior to loading onto SDS gels. After blotting proteins onto PVDF membranes (Immobilon), respective antibodies (see above) were used to confirm co-immunoprecipitation of endogenous WRC subunits with overexpressed EGFP-tagged Sra1.

### Immunofluorescence staining

For immunolabeling of proteins of interest, B16-F1 or NIH 3T3 cells were seeded onto glass coverslips coated with laminin (Sigma) or fibronectin (Roche), respectively. Cells were allowed to adhere overnight. On the following day, cells were fixed with pre-warmed 4% paraformaldehyde (PFA) in PBS for 20 min and permeabilized with 0.05% Triton-X100 (for B16-F1 cells) or 0.1% Triton-X100 (for NIH 3T3 cells) in PBS for 1 min. Prior to antibody staining, cells were blocked with 5% horse serum (Cytogen, 01-06500) in PBS containing 1% bovine serum albumin (BSA; Sigma, A2153) ∼30 min. For sole visualization of the actin cytoskeleton using phalloidin, cells were fixed with 0.25% glutaraldehyde in PBS containing 4% PFA, followed by staining with Alexa Fluor 488-conjugated phalloidin (Invitrogen, A12379, 600 U/ml, 1:200 dilution).

Primary antibodies were diluted in PBS containing 1% BSA and incubated for 1 h. The antibodies used were as follows: anti-ArpC5A (clone 323H3; undiluted hybridoma supernatant; Olazabal et al., 2002), an antibody reactive to FMNL2 and FMNL3 (referred to collectively as FMNL2/3) (Abcam, ab57963; 1:20 dilution), anti-Sra1/PIR121 (#2240; 1:20 dilution; Steffen et al., 2004), anti-Abi (clone W8.3; kindly provided by Dr Giorgio Scita, IFOM, Milano, Italy; undiluted hybridoma supernatant), anti-Nap1 (#2391-C; 1:3 dilution; Steffen et al., 2004), anti-HSPC300 (Abcam, ab87449; 1:100 dilution), anti-pan-WAVE (pAB5502; 1:100 dilution; Schaks et al., 2018), anti-cortactin (Abcam, ab11065-50; 1:100 dilution), anti-lamellipodin (kindly provided by Dr Matthias Krause, King's College, London, UK; 1:200 dilution; Krause et al., 2004), anti-VASP (5500-A; 1:300; Jenzora et al., 2005), anti-myc (Sigma-Aldrich, M5546, clone 9E10; ascites fluid, 1:100 dilution). Mouse primary antibodies were visualized with Alexa Fluor 488-coupled (1:400 dilution) or Alexa Fluor 594-coupled (1:200 dilution) anti-mouse IgG (Invitrogen; #A11029 or #A11032, respectively). Secondary antibodies against rabbit primary antibodies were Alexa Fluor 488-coupled (1:400 dilution) or Alexa Fluor 594-coupled (1:200 dilution) anti-rabbit IgG (Invitrogen, #A11034 or #A11037, respectively). Alexa Fluor 488- and Alexa Fluor 594-coupled phalloidin were obtained from Invitrogen (#A12379 and #A12381, respectively).

### Cell stimulation

For stimulation of cells growing on coated coverslips with serum and growth factors, three washes with PBS were followed by overnight starvation with DMEM lacking any supplements. The next day, cells were treated with prewarmed, regular growth medium for 20 min supplemented with 100 ng/ml hepatocyte growth factor (HGF; Sigma, H5791). Afterwards, cells were fixed and subjected to immunofluorescence staining as described above.

### Random migration assay

For random migration assays, cells were seeded subconfluently into laminin- or fibronectin-coated (for B16-F1 or NIH 3T3 cells, respectively) µ-slide 4-well phase contrast-optimized, glass-bottom microscopy chambers (Ibidi, Martinsried, Germany) using regular growth medium. Ibidi chambers were mounted onto an inverted microscope (for details see next paragraph), equipped with a 37°C incubator and CO_2_ atmosphere as well as an automated stage. Phase-contrast movies were acquired on multiple randomly chosen positions using a 10×/0.15 NA Plan Neofluar objective and a frame rate of 12 frames/h for at least 10 h. For migration speed analyses, cells were manually tracked using ImageJ (https://imagej.nih.gov/ij/), and for determining directionality and mean square displacement of cells, DiPer software was used (Gorelik and Gautreau, 2014).

### Fluorescence recovery after photobleaching

Fluorescence recovery after photobleaching (FRAP) experiments were performed essentially as described previously (Dimchev and Rottner, 2018). In brief, cells were mounted in an open chamber (Warner Instruments) and maintained at 37°C with a heater controller (Model TC-324 B, SN 1176) and appropriate pH using Ham's F12 medium (Gibco, 11765054) containing all growth supplements (4.5 g/l glucose, 10% fetal calf serum and 2 mM L-glutamine). The Axio Observer microscope used was equipped with a DG4 light source (Sutter Instrument) for epifluorescence illumination; a VIS-LED for phase-contrast optics; 63×/1.4 NA or 100×/1.4 NA plan apochromatic objectives for high-magnification microscopy; and a back-illuminated, cooled, charge-coupled-device (CCD) camera (CoolSnap HQ2, Photometrics) driven by VisiView software (Visitron Systems) for image acquisition. Bleaching was performed using a 405 nm diode laser at 60–70 mW output power, which was controlled by the 2D-VisiFRAP Realtime Scanner (Visitron Systems, Germany). Comparison of the turnover of EGFP-tagged Nap1 versus EGFP-tagged Hem1 was performed in the Nap1 KO clone virtually devoid of lamellipodia formation in standard conditions (clone 16). Movies were acquired at ∼2 s per frame (0.5 Hertz). Ectopically expressed, EGFP-tagged proteins were bleached within a lamellipodial region, as indicated in Fig. S4G (red outline). Average intensities in bleached regions were measured over time, and background intensities obtained from extracellular regions were subtracted for each frame. Acquisition photobleaching was eliminated by normalizing data from each time point to non-bleached, lamellipodial tip regions (green outline in Fig. S4G). Data were curve-fitted with SigmaPlot 12.0 (Scientific Solutions SA, Switzerland) using single exponential equation: *f*=*y*_0_+*a*(1−*e*^−*bx*^), and half times of recovery were derived from fitted data.

### Width and F-actin intensity measurements of lamellipodia and LLS

The network width of lamellipodia and LLS was measured using ImageJ (‘measure distance’ plugin) and as illustrated in Fig. 3B (red lines). Actin network density was determined by defining a region (red outlines, Fig. 3B) spanning the lamellipodium or LLS, while avoiding signal contributions from microspikes. Average fluorescence intensities of lamellipodial and LLS actin filaments were determined by measuring average pixel intensities in these regions using the ImageJ ‘measure mean’ plugin. Data are shown as box and whisker plots (Fig. 3C).

### Line scan analyses

Fluorescence intensities of the lamellipodial components ArpC5A (an isoform of ArpC5), cortactin, VASP, Abi, lamellipodin and FMNL2/3 (Figs 3 and 4) were measured by drawing an orthogonal line (3 µm long, 5 pixels wide) across the cell edge. Line scan graphs represent corresponding average intensities ranging from the cell center (left) to the outside (right). Line scan measurements were performed using MetaMorph software (Molecular Devices, USA) and further processed using Microsoft Excel. At least 30 cells for each cell clone and distinct staining were analyzed and averaged. Background intensities outside the cell were subtracted, values were normalized according to the maximal/minimal intensity measured for each condition and plotted over the entire line distance of 3 µm. Due to this procedure, line scan measurements can only give relative information for each component concerning the distribution of fluorescence signal along the scan, but do not provide any quantitative comparisons between distinct components, neither in the same cells and measured regions, nor in different cell types.

### 3D structured illumination microscopy

Images were acquired on a Nikon SIM-E super-resolution microscope equipped with a LU-N3-SIM 488/561/640 laser unit mounted on a Nikon Ti Eclipse (Nikon) and a Piezo *z* drive (Mad city labs). Images were taken using a CFI Apochromat TIRF 100×/1.49 NA oil immersion objective (Nikon), a Hamamatsu Orca flash 4.0 LT camera and an N-SIM motorized quad band filter combined with N-SIM 488 nm and 561 nm bandpass emission filters using the 488 nm laser line driven by NIS-Elements software (Nikon). Reconstructions were performed employing the slice reconstruction tool (Nikon, NIS-Elements).

### *Listeria* invasion assay

Bacterial invasion with *L. monocytogenes* (EGD wild-type strain) and isogenic, specific invasion-deficient control bacteria (ΔInlA/B; Parida et al., 1998) into wild-type NIH 3T3 and corresponding Nap1/Hem1 double KO clones, or Rac1^flox/flox^ control and corresponding Rac1 KO clone 3 fibroblasts (Steffen et al., 2013), was quantified by gentamycin protection assay, which was performed essentially as described previously (Bosse et al., 2007). The only exception was that as opposed to previous protocols (Elsinghorst, 1994), host cell stress was minimized by performing infections in the presence of DMEM containing 2% fetal calf serum (FCS, Sigma) as opposed to DMEM alone. Data from three and four independent experiments for Rac1 KO and Nap1/Hem1 double KO clones, respectively, were displayed as normalized to 100% of wild-type host cell control in each case, and subjected to statistical analyses as specified below.

### Scanning electron microscopy

For the visualization of invasion events with *L. monocytogenes*, we used a modified version of a previously described assay (Rohde et al., 2003). In brief, NIH 3T3 control (5×10^4^ cells) or Nap1/Hem1 double KO clones (1×10^5^ cells) were seeded onto 12 mm coverslips in wells of a 24-well plate and allowed to grow for 18–20 h before infections. Infections were performed with *L. monocytogenes* diluted 1:100 into DMEM containing 2% FBS after overnight culture in BHI medium (brain heart infusion broth, BD, 237500, 37 g/l in H_2_O) and a gentle washing step in DMEM. Invasion was stopped at various time points after start of infections by adding fixative to the growth medium (final concentrations of 5% formaldehyde and 2.5% glutaraldehyde) in order to maximize the probability of capturing active entry events. After fixation, coverslips were washed twice with TE buffer (20 mM Tris-HCl, 1 mM EDTA, pH 6.9) and dehydrated in 10 min steps at increased acetone concentrations on ice (10, 30, 50, 70, 90%), followed by two steps using 100% acetone and room temperature. Cells were critical-point dried with liquid CO_2_ (CPD 30, Balzers, Liechtenstein) and covered with a palladium–gold film by sputter coating (SCD 500, Bal-Tec, Liechtenstein) before examination in a field emission scanning electron microscope (Zeiss Merlin, Oberkochen, Germany) using the HESE2 Everhart Thornley SE detector and the in-lens SE detector in a 25:75 ratio at an acceleration voltage of 5 kV.

### Data processing and statistical analyses

Brightness and contrast levels of images were adjusted using MetaMorph software. Figures were further processed and assembled with Photoshop CS4. Data analyses were carried out in ImageJ and MetaMorph, Excel 2010 and Sigma plot 12.0. Statistical comparisons of multiple datasets were done using one-way ANOVA with post-hoc Dunnett's test (Graphpad Prism 6.01). In case of pairwise comparisons, non-parametric Mann–Whitney rank sum test was used (Sigma plot 12.0). A probability of error of 5% (*P*≤0.05; * in figure panels) was considered to indicate statistical significance. **, *** and **** indicate *P*-values of ≤0.01, ≤0.001 and ≤0.0001, respectively.

## Supplementary Material

Supplementary information

Reviewer comments
