# Supplementary material for: Lamellipodia-like actin networks in cells lacking WAVE regulatory complex
Source: J Cell Sci. 2022 Aug 16;135(15):jcs260364. doi: 10.1242/jcs.260364 (PMC9511706; doi:10.1242/jcs.260364)
Supplement: Supplementary information [file joces-135-260364-s1.pdf]

Figure S1

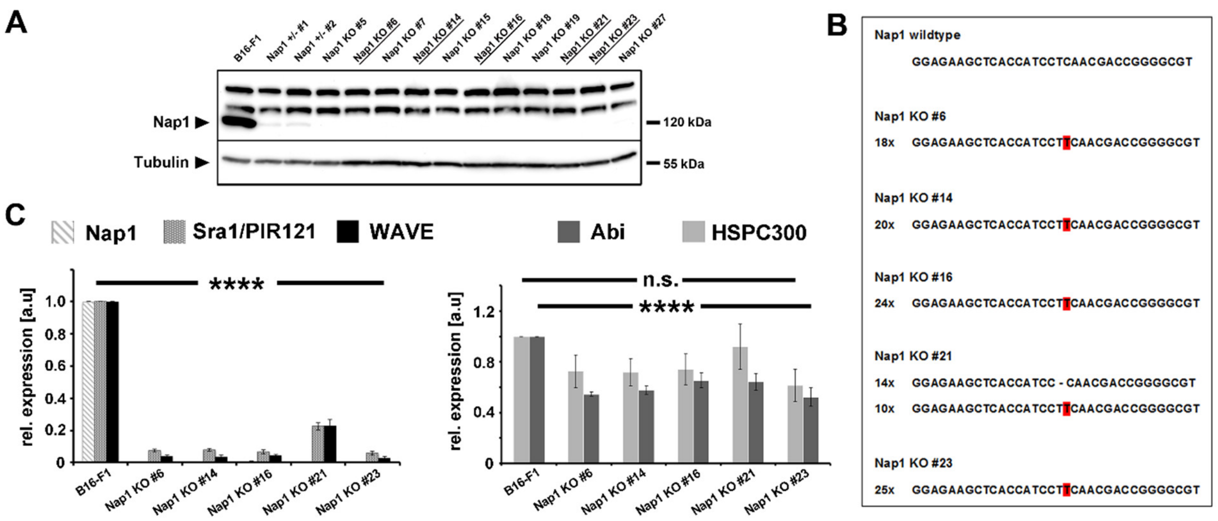

Fig. S1. Genetic removal of Nap1 alters expression levels of remaining WRC subunits. (A) CRISPR/Cas9-mediated loss of Nap1 expression as documented by Western blotting. Two clones (#1 and #2) harbored a faint, residual expression of Nap1 protein, but clones #6, #14, #16, #21 and #23 were completely devoid of detectable Nap1 expression and selected for further analyses (underlined). α-Tubulin was probed for equal sample loading. **(B)** Sequencing results of the CRISPR/Cas9-modified Nap1 gene locus (exon 1) of respective cell clones. Numbers on the left illustrate the frequencies of given mutations found, indicating that all clones aside from clone #21 likely possess the same insertion on all alleles present. Yet, all alleles were confirmed to result in frame-shifts and premature stop-codons (not shown). **(C)** Quantitation of expression levels of WRC subunits in Nap1 KO clones as compared to B16-F1 wildtype cells. Bar charts represent relative protein expression measured on ECL exposed membranes normalized to Tubulin from 4 independently generated lysates for each clone. Error bars are ± sem. Note that loss of Nap1 is accompanied by significant reduction of Sra1/PIR121 and WAVE levels, while Abi and HSPC300 levels are less affected (the latter found not to be statistically significant throughout all clones). Statistics were done using one-way ANOVA with Dunnet's adjustment for multiple comparisons; **** = $p \leq 0.0001$, n.s (not significant) = $p \geq 0.05$.

Figure S2

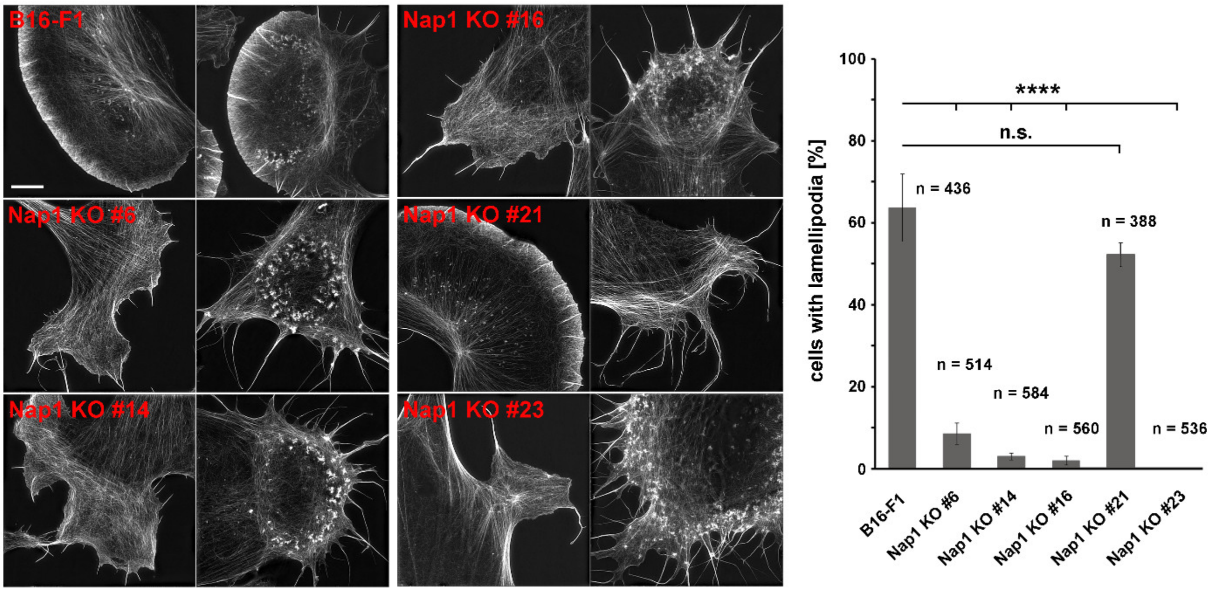

Fig. S2. Nap1-depleted cells can still form lamellipodia. Structured Illumination Microscopy (SIM) images of different Nap1 KO clones and B16-F1 control cells (two representative images each) stained for the filamentous actin cytoskeleton (F-actin). More than 60% of wildtype B16-F1 cells form lamellipodia on laminin. Unexpectedly, subfractions 2 of Nap1 KO clones are still capable of forming lamellipodia (left example for all clones), whereas the majority of cells extend filopodia and harbor actin-rich clusters near cell peripheries (examples on the right). Note that lamellipodia in Nap1 null clones are commonly thinner and less F-actin-dense, except for those observed in Nap1 KO #21. Bar graph on the right shows the percentage of cells harboring lamellipodia structures independent of their morphology. Data in the bar chart are arithmetic means ± sem from three independent experiments. One-way ANOVA with Dunnet's adjustment for multiple comparisons; **** = p ≤ 0.0001 and n.s. = p ≥ 0.05.

Figure S3

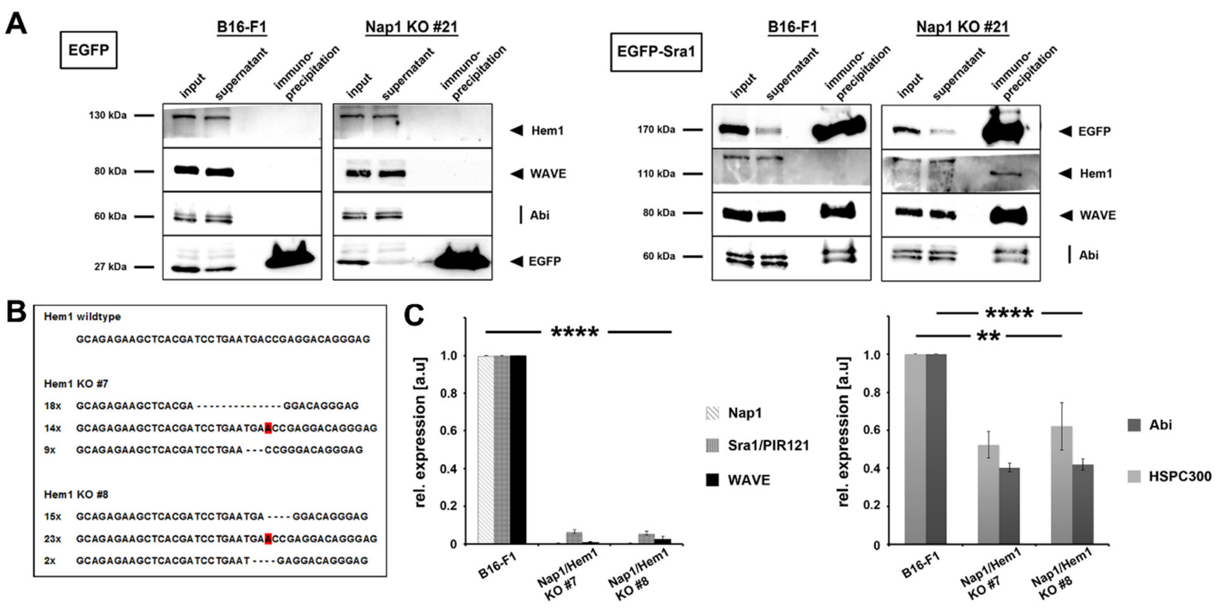

Fig. S3. Nap1 disruption can cause compensatory upregulation of Hem1 expression. (A) Co-immunoprecipitation of endogenous WRC subunits including Hem1 with EGFP-tagged Sra1 in Nap1 KO #21 (right panel), but not in B16-F1 cells or in either cell line using EGFP alone as control (left panel). Note that endogenous WRC subunits are also robustly precipitated in B16-F1 wildtype cells (exemplified by WAVE and Abi), but as opposed to Nap1 KO #21 (right panel), those precipitates lacked Hem1, likely due to lack of detectable Hem1 expression in B16-F1 wildtype cells. **(B)** Genomic DNA sequencing of the *hem1* locus upon generation of Nap1/Hem1 double KO cell lines (double KO clones #7 and #8, as indicated), revealing various mutations in the *hem1* gene locus (exon 1). In both clones, three distinct mutations on apparently at least three distinct alleles were found, most of which caused premature stop signals. The in-frame deletion (3 nucleotides) found on one allele present in Nap1/Hem1 KO clone #7 was confirmed to represent a loss-of-function mutation (see Fig. S5). **(C)** Quantitation of expression of remaining WRC subunits in B16-F1 wildtype *versus* Nap1/Hem1 double KO clones #7 and -#8, derived from 6 independently generated cell extracts. Although suppression of remaining WRC subunit expression was clearly differential, reduction was statistically significant for all subunits. One-way ANOVA with Dunnet's adjustment for multiple comparisons; **** = p ≤ 0.0001, ** = p ≤ 0.01.

Journal of Cell Science • Supplementary information

Figure S4

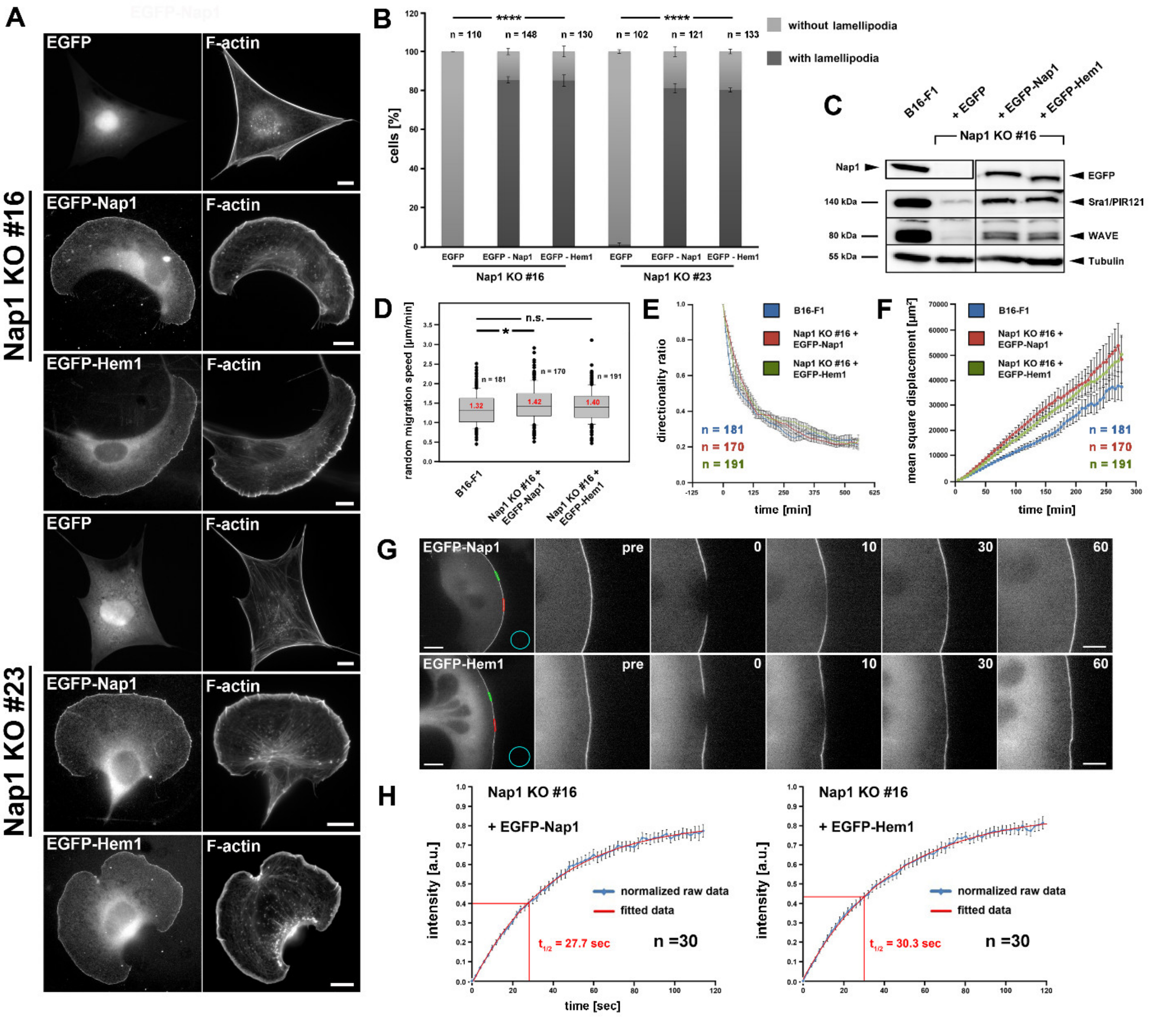

Fig. S4. Nap1 and Hem1 have redundant functions in lamellipodia formation and cell migration. (A) Comparison of lamellipodia formation rescued by transient expression of either EGFP-Nap1 or -Hem1 in two clones virtually devoid of lamellipodia formation (B16-F1 clones #16 and #23). Images display EGFP fluorescence of respective construct and the actin cytoskeleton counterstained with phalloidin. Scale bars, 10 µm. **(B)** Quantification of cells harboring lamellipodia upon expression of constructs as in A. Data in the bar chart are arithmetic means ± sem from three independent experiments. Number and morphology of such reconstituted lamellipodia were indistinguishable between Nap1 and Hem1-expressing cells. **(C)** Cell lysates of Nap1- or Hem1-transfected cells compared to EGFP-expressing KO

cells or wildtype B16-F1. Expression of EGFP-Nap1 versus EGFP-Hem1 confirmed equal rescue of endogenous WRC subunit expression (Sra1/PIR121 and WAVE). α-Tubulin was used as loading control. **(D)** B16-F1 control as well as Nap1 KO cells re-expressing either Nap1 or Hem1 were analyzed for random migration speed and data are shown as box and whisker plots. Statistics in B and D were done using one-way ANOVA with Dunnet's adjustment for multiple comparisons; **** = $p \leq 0.0001$, * = $p \leq 0.05$ and n.s. = $p \geq 0.05$. **(E)** Graph displaying changes in migration directionality over elapsed time (min) for cell lines as illustrated by the color code. Directionality is displayed as the ratio of displacement to trajectory length for each time point. Error bars are ± sem. **(F)** Mean square displacement (μm^2) over elapsed time (min) for indicated cells lines. Error bars for each time point are ± sem. Note the marked but highly comparable increase of MSD for cell populations expressing EGFP-tagged Nap1 *versus* -Hem1. **(G)** Time-lapse frames of Nap1- or Hem1-expressing cells subjected to FRAP. Time is given in seconds. First image illustrates individual regions that were used for bleaching of fluorescent protein at the lamellipodium tip (red outline), for determination of acquisition photo-bleaching in a reference region (green outline) and background intensity (cyan circle). Bars are 10 µm in overviews and 3 µm in insets. **(H)** FRAP analysis of either EGFP-Nap1 or -Hem1 expressed in Nap1 KO cells. Raw data (blue curve) are plotted as arithmetic means ± sem of fluorescence intensities at acquired time points after bleaching. Half-times of recovery (red numbers) for each component were derived from respective curve fits (red lines).

Figure S5

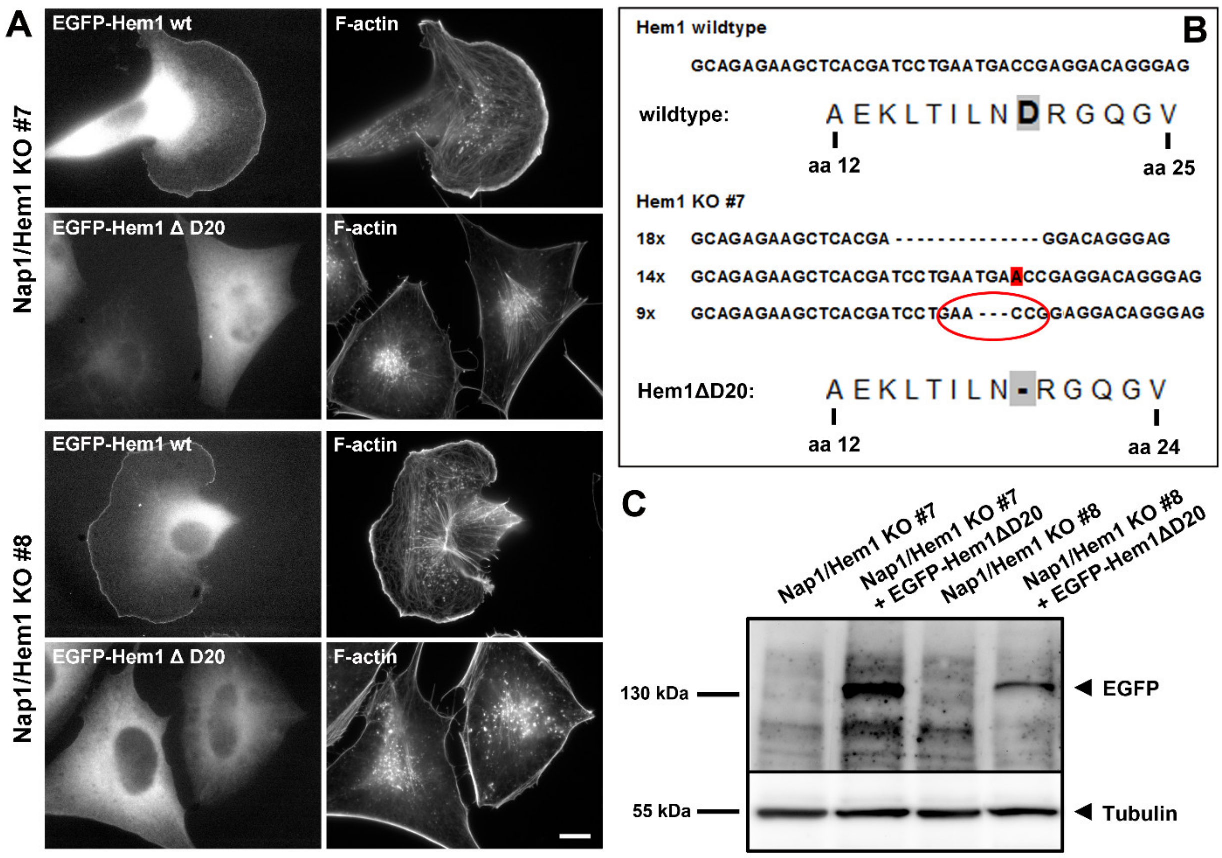

Fig. S5. A single Hem1 amino acid depletion disrupts its lamellipodia formation capability. (A) Nap1/Hem1 CRISPR/Cas9-treated clones were probed for rescue of lamellipodia formation with EGFP-tagged, wildtype Hem1 or an in-frame deletion mutant lacking aspartate at position 20, as endogenously observed - among other variants - in Nap1/Hem1 double KO #7 (see Fig. S3B). While wildtype Hem1 efficiently rescued lamellipodia formation in both cell lines, as confirmed by F-actin staining using phalloidin (right panels), mutated Hem1ΔD20 completely failed to induce lamellipodia protrusions. Scale bar, 10 µm. **(B)** Comparison of genomic sequences and their translated amino acid residues in the relevant region of exon 1 in wildtype *versus* Nap1/Hem1 KO #7, which is devoid of lamellipodia formation in standard conditions (see Fig. 1F). Note that the in-frame deletion in Nap1/Hem1 double KO #7 among other alleles causes the loss of aspartate at position 20 (Hem1ΔD20). **(C)** Western blot using an EGFP-specific antibody to confirm expression of the Hem1ΔD20-construct as full length protein at expected molecular weight, excluding that its lack of capability to mediate lamellipodia formation is caused by failure of proper expression. α-Tubulin was used as loading control.

Journal of Cell Science • Supplementary information

Figure S6

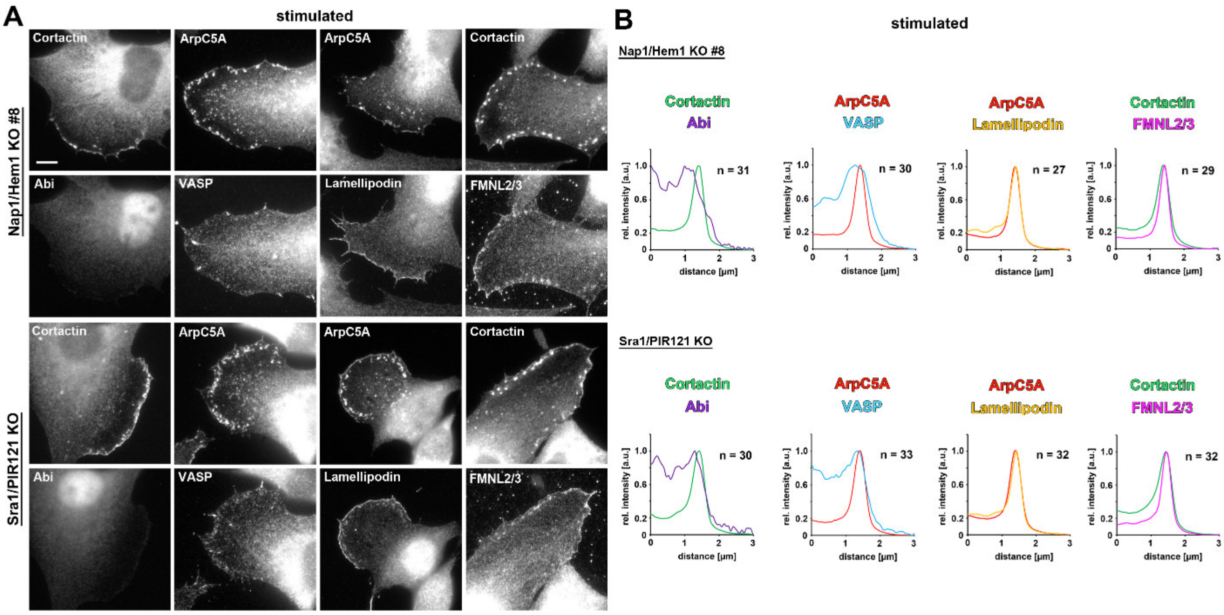

Fig. S6. LLS are enriched for canonical lamellipodial tip markers Lamellipodin and FMNL2/3, but not for VASP. (A) Starved and stimulated (HGF in full medium) cells harboring genotypes as denoted on the left. Cells harboring lamellipodia-like structures (LLS) were stained for various lamellipodial markers as indicated, and co-stained for either ArpC5A (monoclonal) or Cortactin (polyclonal), dependent on the species of respective, combined antibody. Note that VASP is enriched in peripheral focal adhesions, but absent from the tips of LLS in WRC-depleted cells. Scale bar, 10 µm. **(B)** Line scan measurements as in Fig. 4B.

Journal of Cell Science • Supplementary information

Figure S7

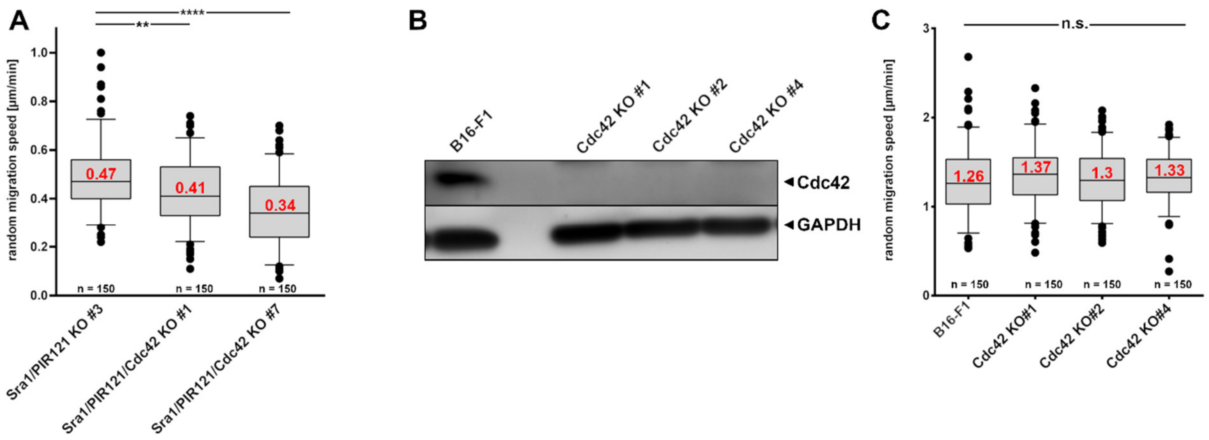

Fig. S7. Cdc42 removal reduces migration of B16-F1 cells in the absence of WRC, but not in its presence. (A) Random migration assay using combined WRC and Cdc42 KO cells established and characterized in Fig. 6 as compared to their parental, control cell line lacking WRC alone (Sra1/PIR121 KO #3). Both clones, additionally eliminated for Cdc42 expression, display migration rates that were reduced in a statistically significant fashion. **(B)** Western blotting confirming the loss of Cdc42 expression upon CRISPR/Cas9-mediated targeting of the *Cdc42* locus in the wildtype background (three independent Cdc42 KO clones analyzed, as indicated). **(C)** Random migration efficiency of B16-F1 wildtype *versus* Cdc42 KO cells, confirming that Cdc42 loss of function does not significantly impact on migration rates in the presence of WRC. Detailed characterization of the new Cdc42 KO lines will be described elsewhere. Statistics in A and C were done using one-way ANOVA with Dunnet's adjustment for multiple comparisons; **** = $p \leq 0.0001$, ** = $p \leq 0.01$, n.s (not significant) = $p \geq 0.05$.

Journal of Cell Science • Supplementary information

Figure S8

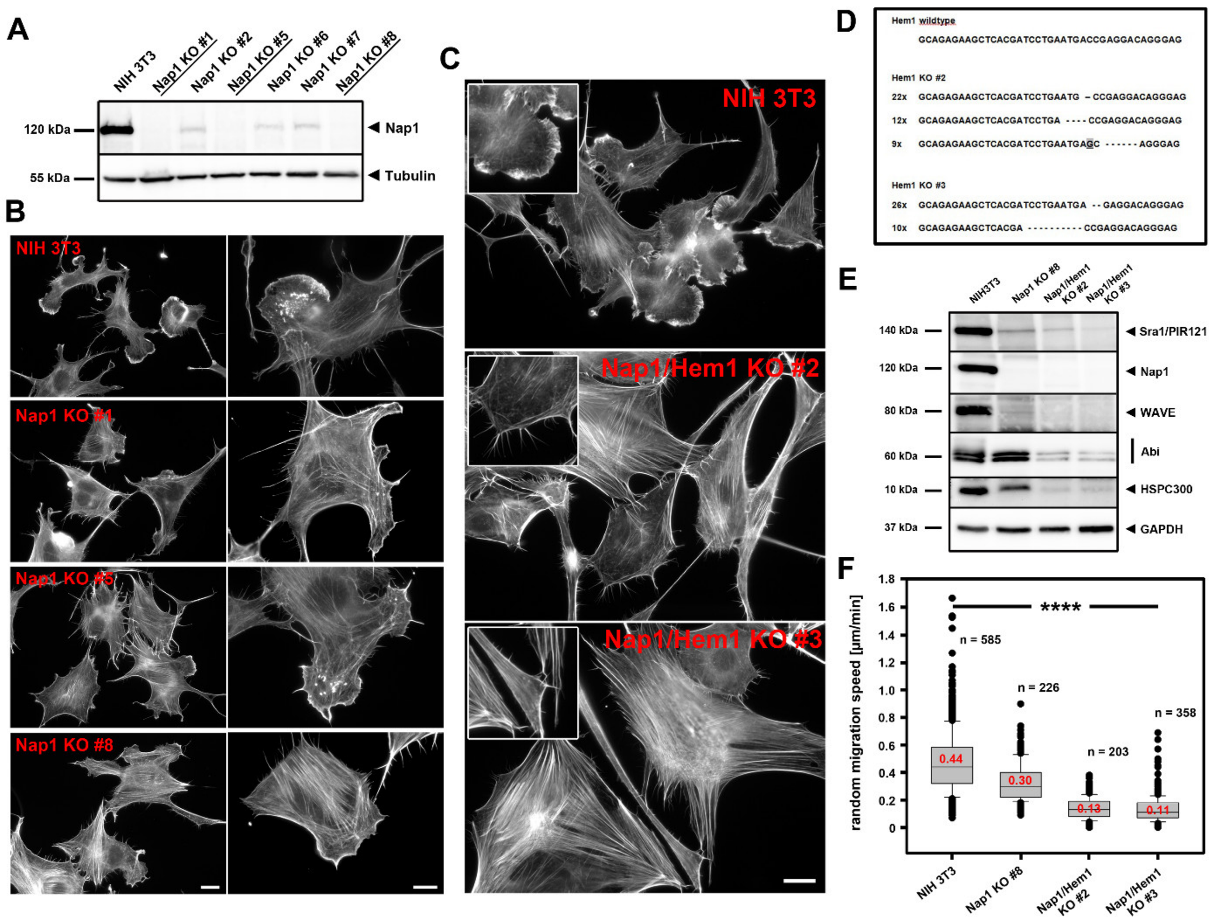

Fig. S8. Generation and characterization of Nap1 single and Nap1/Hem1 double KO fibroblast cell lines. (A) Anti-Nap1 western blot confirming CRISPR/Cas9-mediated deletion of Nap1 expression in NIH 3T3 fibroblasts (underlined, clones #1, #5, #8). **(B)** Representative, phalloidin-stained cell images showing Nap1 KO cells forming lamellipodia, although these structures possess compromised morphologies. Overview images left column (scale bar, 20 µm); higher magnifications right (scale bar, 10 µm). **(C)** Nap1-deficient clone #8 was subjected to additional, CRISPR-mediated Hem1 removal, causing complete loss of lamellipodia protrusions and increased stress fiber formation in Nap1/Hem1 double KO cell lines as compared to NIH 3T3 wildtype in standard growth conditions (insets show close-ups). Scale bar, 20 µm. **(D)** Sequencing data information from the *hem1* gene locus for the two analyzed clones (Nap1/Hem1 KO #2 and #3). **(E)** Western blot analyses of WRC subunit expression in NIH 3T3 wildtype, Nap1 KO #8 and Nap1/Hem1 double KO clones, as indicated. Note dependence of WRC subunit expression on Nap1/Hem1 gene dose, as illustrated by abrogated Abi1 or HSPC300 expression only in Nap1/Hem1 double KO cell lines. **(F)** Box and whisker plot representing random migration speed of cell lines as denoted in the figure.

Journal of Cell Science • Supplementary information

Table S1. Table summarizes various cell lines **(genotypes, 1. column)** that have been used throughout this study. These cell lines were either untreated (X), subjected to growth factor stimulation (stimulated) or transfected with certain plasmids as indicated **(treatment, 2. column)**. Upon subjecting to these different experimental conditions, cells either formed lamellipodia **(lamellipodia, 3. column)** or lamellipodia-like structures **(LLS, 4. column)** with certain frequencies given as percentages in respective columns. Depending on the column, X either indicates that cells were untreated or incapable of forming lamellipodia/LLS. The last column refers to related figures **(related figure, 5. column)** that contain the underlying data.

genotype	*treatment*	*lamellipodia*	*LLS*	*related figure*
**B16-F1 wildtype**	X	~ 64 %	X	Fig. S2
**Nap1 KO #6**	X	X	~ 9 %	Fig. S2
**Nap1 KO #14**	X	X	~ 3 %	Fig. S2
**Nap1 KO #16**	X	X	~ 2 %	Fig. S2
**Nap1 KO #21**	X	~ 52 %	X	Fig. S2
**Nap1 KO #23**	X	X	< 1 %	Fig. S2
**Nap1 KO #21-12**	X	Frequency not determined, but indistinguishable from WT	X	Fig. 1D
**Nap1/Hem1 KO #7**	X	X	X	Fig. 1F
**Nap1/Hem1 KO #8**	X	X	X	Fig. 1F
**Nap1/Hem1 KO #7**	stimulated	X	~ 7 %	Fig. 3A
**Nap1/Hem1 KO #8**	stimulated	X	~ 7 %	Fig. 3A
**Sra1/PIR121 KO**	stimulated	X	~ 10 %	Fig. 3A
**Rac1/2/3 KO**	stimulated	X	X	Fig. 3A
**B16-F1 wildtype**	+ Cdc42-L61	100 %	X	Fig. 6A,C
**Sra1/PIR121 KO**	+ Cdc42-L61	X	~ 22 %	Fig. 6A,C
**Sra1/PIR121 KO + N-WASP KO #5**	+ Cdc42-L61	X	X	Fig. 6A,C
**Sra1/PIR121 KO + N-WASP KO #9**	+ Cdc42-L61	X	X	Fig. 6A,C
**Sra1/PIR121 KO**	stimulated	X	~ 11 %	Fig. 6D
**Sra1/PIR121 KO + Cdc42 KO #1**	stimulated	X	X	Fig. 6D
**Sra1/PIR121 KO + Cdc42 KO #7**	stimulated	X	X	Fig. 6D
**Sra1/PIR121 KO**	+ Cdc42-L61	X	~ 14 %	Fig. 6E,F
**Sra1/PIR121 KO**	+ Cdc42-L61/A37	X	~ 2%	Fig. 6E,F
**Sra1/PIR121 KO**	+ Cdc42-L61/C40	X	X	Fig. 6E,F

Journal of Cell Science • Supplementary information
