# Supplementary material for: Lamellipodia-like actin networks in cells lacking WAVE regulatory complex
Source: J Cell Sci. 2022 Aug 16;135(15):jcs260364. doi: 10.1242/jcs.260364 (PMC9511706; doi:10.1242/jcs.260364)
Supplement: Reviewer comments [file joces-135-260364_review_history.pdf]

Lamellipodia-like actin networks in cells lacking WAVE regulatory complex

Frieda Kage, Hermann Döring, Magdalena Mietkowska, Matthias Schaks, Franziska Grüner, Stephanie Stahnke, Anika Steffen, Mathias Müsken, Theresia E. B. Stradal and Klemens Rottner
DOI: 10.1242/jcs.260364

Editor: Michael Way

Review timeline
Submission to Review Commons: 18 June 2021
Original submission: 21 June 2022
Editorial decision: 22 June 2022

Reviewer 1

Evidence, reproducibility and clarity

The study by Kage et al. addresses the molecular mechanisms required for the formation of Rac-dependent lamellipodia, by disrupting the subunit Nap1 and its paralogue Hem1 of the WRC complex. The authors identify in this way a pathway for the formation of Arp2/3- dependent lamellipodia-like structures that require Rho family GTPases but that are independent on WRC. The study is potentially interesting, but a number of important points need to be addressed to improve the clarity of the information and interpretation of the data.

The data presented in Figures 1 and 2 convincingly show that double KO of Nap1 and Hem1 subunits, heavily affects lamellipodia formation, with rescue experiments supporting the requirement of these subunits, and therefore of a functional WRC complex for the formation of lamellipodia. In Figure 1, quantification of the percentage of cells with lamellipodia in different clones/experimental conditions would make the description of the results easier to follow. In Figure 1D, the panel for Nap1 for clone #21 appears to show still a diffuse signal for this subunit.

On the other hand the authors mention their surprise in finding that growth factors induce what they call lamellipodia-like structures (LLS). Why do authors define these as lamellipodia-like structures: what is the difference according to them between lamellipodia and LLS? This should be clearly explained somewhere in the text. And how do the authors explain the lack or presence of lamellipodia in the same cells but distinct experimental conditions?

Also, what would be really helpful to the reader is a schematic drawing with the network(s) they are referring to in different experimental settings described in the paper. In this respect, it is sometimes difficult to follow the author's reasoning and deductions from their data; the drawing(s) would make their interpretations more clear. In some cases also rewriting is needed, to expose the concepts in a clearer, more explicit manner (see as one example the discussion at page 10, lines 16-19).
Reference to models would also help understanding the interpretation of the data in support of a WRC-independent mechanism for the formation of lamellipodia.

Figure 4 and S5, authors mention that VASP is an exception (page 10); I guess they refer to the fact that VASP is NOT accumulated at the cell edge (Figure 4); but then, VASP is clearly accumulated at adhesions at the cell edge in the same dKO clones (Figure S5). The conclusion at page 10 is not supported by the images shown in Figure S5. This is confusing and should be explained clearly. Considering also what authors describe in the case of VASP in cells overexpressing C.A. Cdc42

© 2022. Published by The Company of Biologists under the terms of the Creative Commons Attribution License (https://creativecommons.org/licenses/by/4.0/).

(Figure 5), it looks like authors refer to more subtle differences concerning the distribution of VASP at the edge (continuous line vs focal points). Moreover, in Figure 5 it is not sufficient to refer to a previous paper for control distribution of VASP: WT cells should be included in this figure as proper control from the same experiments as for KO clones.
The reason of using the C.A. GTPAse is not quite clear here. It is well known that C.A. GTPases may induce phenotypes deriving from an altered functional cycle, which is not physiological. What is the rationale for showing these experiments? and the relationship to what has been discussed earlier? This is not clear from the way the data are presented. And once more, a reference scheme would help.

Significance

The study by Kage et al. addresses the molecular mechanisms required for the formation of Rac-dependent lamellipodia, by disrupting the subunit Nap1 and its paralogue Hem1 of the WRC complex. The authors identify in this way a pathway for the formation of Arp2/3- dependent lamellipodia-like structures that require Rho family GTPases but that are independent on WRC.

Reviewer 2

Evidence, reproducibility and clarity

The manuscript by Kage et al. describes a highly original study of novel lamellipodia-like structures (LLS), which are independent of the WAVE regulatory complex (WRC), in mouse melanoma and fibroblast cell lines in culture. First, the authors have observed that a KO of a key WAVE subunit, NCKAP1, did not abolish the formation of lamellipodia in all clones they have obtained (an exact quantification is necessary there (1). They have concluded that an up-regulation of Hem1/NCKAP1L is responsible for the lamellipodia in some of their clones, and have indeed shown that Hem1 was present, by having inserted a GFP tag before Hem1 in the genome (2).

The authors then moved on to inactivate both NCKAP1 and Hem1 in B16-F1 cells, and to study the cell motility and formation of protrusions. These cells, when starved and then stimulated with growth factors, were capable of making "smaller than lamellipodia" (3) LLSs. These structures were observed in all WRC-depleted cell lines, but not in RAC1-3 KO cells. The authors show that WAVE subunits were not present in LLS, though the data are not quite clear (4).

Then, the expression of Cdc42 in double KO NCKAP1/Hem1 cells is shown to induce the LLS (5). Finally, the authors conclude that these ARP2/3 containing structures can exist in B16-F1 or NIH 3T3 mouse cells, independently of WRC, but in direct link with CdC42 and/or Rac stimulation (6).

(1). In multiple experiments shown in the article, the occurrence of cells is described as "occasional" or "frequent". An exact quantification is necessary, to be able to evaluate these results. In each case of a "typical field", it will be very helpful to have the exact percentage of cells in question in a larger population.

(2) It is hardly a conventional method, to tag an endogenous protein by GFP in order to show its expression. Why did not a simple western blot work? At least a comment about the choice of this technique is necessary here. What is the level of Hem1 expression, compared to the original NCKAP1 expression? The GFP KI approach can help to answer this important question.

(3) A quantification is needed here, because it is not clear what the authors mean by these novel structures being "smaller" than the lamellipodia.

(4) In Fig.4, upper panels, the quantification of ABI1 localisation rather gives an impression that ABI1 (and VASP, as well) is present in the cell body AND at the protrusions, contrary to other factors that are specifically enriched at the LLS edge, but not in the cell body. This does not mean that ABI1 and VASP are absent at the edge.

(5) It will be useful to clearly define "LLS", and also to quantify the frequency of their appearance in this experiment, as well.

© 2022. Published by The Company of Biologists under the terms of the Creative Commons Attribution License (https://creativecommons.org/licenses/by/4.0/).

(6) The interplay between the Rac family and Cdc42 in this study is completely unclear. The authors show that Rac KO cells never form the LLS, and that WRC-depleted cells form the LLS when overexpressing Cdc42. Can these factors compensate for each other? More experiments are necessary to illustrate this question, and a model will also be very useful, in order to fully understand the proposed hypothesis. It will also be very helpful if the authors could suggest a possible mechanism of Rac/CdC42-dependent mode of WRC-independent activation of ARP2/3 in the LLSs, at least as a model.

Significance

This is a novel and interesting study, and I hope that it will be published soon. However, more precision, quantification and statistical analysis, and some additional experiments are needed, as well as some editorial work, in order to make the story clear and straightforward.

Referee Cross-commenting

I fully agree with the comments of Reviewer 1, especially with regard to the absence of a clear model for the proposed LLS protrusions (such model(s) should be included in the paper), and the contradictions concerning VASP localisation.

With regard to Reviewer 3, I do agree with the majority of the comments, except that I find that the manuscript represents potentially interesting and novel data, and not just a confirmation of findings from other groups. However, it is not ready to be published in its current form.

Reviewer 3

Evidence, reproducibility and clarity

Kage et al

In this study, the authors explore the types of actin protrusions that form in the absence of the Wave Regulatory complex (WRC). They conclude that small, Arp2/3 containing protrusions, that they call lamellipodia like structure, still form in cells where WRC components have been eliminated by CRISPR-Cas9-based gene knockout. These LLS structures require CDC42 and N-WASP. Somehow Rac is still required as well, but explanation for this result is given. Finally, there is a marked decrease in Ena/VASP localization to the leading edge upon WRC deletion. For the most part, the experiments use standard techniques and are well executed. The problem with this paper is not in the evidence presented, but rather that it is all just confirmatory of findings from other groups. Very little new insight into the formation of actin protrusions is presented (more on that below). A few specific issues with the data in the paper:

1. The VASP staining in the Nap/Hem knockout is not really completely missing from the leading edge, but rather reduced based on what I see in the images (Sup Fig 5). The WT cells have a much brighter band of staining, but this is likely a mixture of leading edge staining at the tip of the lamellipodia and a row of nascent adhesions just behind it.
2. The significance of D21 in Hem1 is not clear to me. There is a known structure that could be consulted
3. The Listeria experiments come completely out of the blue in the paper. They are not mentioned in the abstract, introduction or concluding remarks.
4. The requirement for Rac, even in the absence of the WRC, is interesting, but the authors do nothing to explore this or even speculate about a possible mechanism. This was disappointing.

Significance

This paper is not a very significant advance for the field (one in which I work directly). For example, we know that Nap1 and Hem1 are functionally redundant proteins, therefore is not surprising that if you have a clone where you knockout Nap1 and Hem1 is upregulated that these cells would have proper lamellipodia. We know that Ena/VASP proteins interact directly with WRC, therefore the reduction of localization of VASP upon WRC deletion is not surprising. Recent

© 2022. Published by The Company of Biologists under the terms of the Creative Commons Attribution License (https://creativecommons.org/licenses/by/4.0/).

published work confirms this in C. elegans (PMID: 34281597). The upregulation of N-WASP to compensate for loss of Wave/Scar is known in other systems.

Author response to reviewers' comments

Point-by-point reply to reviewers' comments: To aid readability, reviewers' comments are provided in *Italic*, and our responses in **bold**.

Reviewer 1:

(Evidence, reproducibility and clarity (Required)):

The study by Kage et al. addresses the molecular mechanisms required for the formation of Rac-dependent lamellipodia, by disrupting the subunit Nap1 and its paralogue Hem1 of the WRC complex. The authors identify in this way a pathway for the formation of Arp2/3- dependent lamellipodia-like structures that require Rho family GTPases but that are independent on WRC.

The study is potentially interesting, but a number of important points need to be addressed to improve the clarity of the information and interpretation of the data.

We would like to thank the reviewer for his/her kind words.

The data presented in Figures 1 and 2 convincingly show that double KO of Nap1 and Hem1 subunits, heavily affects lamellipodia formation, with rescue experiments supporting the requirement of these subunits, and therefore of a functional WRC complex for the formation of lamellipodia. In Figure 1, quantification of the percentage of cells with lamellipodia in different clones/experimental conditions would make the description of the results easier to follow. In Figure 1D, the panel for Nap1 for clone #21 appears to show still a diffuse signal for this subunit.

We can see the point concerning quantification, so are providing this information now in new Figure S2, where the reviewer and reader can compare lamellipodia formation frequencies in nap1 null clones harbouring differential amounts of Hem1, so still WRC- dependent structures. This confirms how dramatically clone #21 sticks out, not only concerning overall lamellipodia morphology (left panel), but also concerning the frequency of their formation.

We respectfully disagree though with the second point of the reviewer. The Nap1 band is completely gone in all clones (including clone #21). The reviewer has probably mixed this up with the very faint WAVE band just below (at 85kDa), which is still detected at very low but specific levels in the absence of Nap1, but the Nap1 signal is the same in all clones and completely zero, just constituting background.

On the other hand the authors mention their surprise in finding that growth factors induce what they call lamellipodia-like structures (LLS). Why do authors define these as lamellipodia-like structures: what is the difference according to them between lamellipodia and LLS? This should be clearly explained somewhere in the text. And how do the authors explain the lack or presence of lamellipodia in the same cells but distinct experimental conditions?

We have explained this better now in the revised manuscript version, and apologize for any confusion caused. In brief, in normal growth conditions (such as full serum without supplemented growth factors in B16-F1 or NIH3T3 fibroblasts), WRC is completely dominant and essential for lamellipodia formation. This also means that we believe in a wildtype cell in normal growth conditions, lamellipodia formation through WRC will always dominate and/or override any alternative pathway, which is much less efficient.

However, in more stimulatory conditions, such as upon growth factor stimulation, challenge with infective bacteria or constitutive activation of Cdc42, we can trigger additional contributions to lamellipodia protrusion, which are difficult to discern in the presence of

© 2022. Published by The Company of Biologists under the terms of the Creative Commons Attribution License (https://creativecommons.org/licenses/by/4.0/).

WRC, but which can enhance the frequency of lamellipodia formation in wildtype cells up to 100%, just as example. In the absence of WRC, the same mechanism can trigger the formation of what we define as lamellipodium-like structure, because this structure is thinner and smaller in dimension as compared to a normal lamellipodium in a wildtype cell, but still harbours an Arp2/3 complex-containing network at the cell periphery with all canonical lamellipodial factors except for VASP. Of course, we have wondered: How are we supposed to call these structures? How do we define a lamellipodium, except for being a flat, thin sheet of cytoplasm filled with an actin network and protruding at the periphery of cells growing on flat surfaces? I would say the community would currently agree on lamellipodia comprising networks of actin filaments harbouring dendritic arrays of at least Arp2/3 complex and formed downstream of Rac small GTPases. Like lamellipodia, LLS require Rac and harbour Arp2/3 complex, so what justifies not calling them a lamellipodium? They do harbour any other lamellipodial component though, except for VASP. However, since Ena/VASP turned out not to be entirely essential for lamellipodia protrusion (see lamellipodia formed in Ena/VASP triple KO cells, PMID: 32391788), and since LLS were not comparable in dimension, F-actin intensity and frequency of formation (see new quantitations in Fig. 3A-C) to lamellipodia formed in wildtype cells, we came to the conclusion that LLS would be the most appropriate term to describe those structures lacking both WRC and Ena/VASP.

Also, what would be really helpful to the reader is a schematic drawing with the network(s) they are referring to in different experimental settings described in the paper. In this respect, it is sometimes difficult to follow the author's reasoning and deductions from their data; the drawing(s) would make their interpretations more clear.

We would like to thank the reviewer for this very constructive suggestion - a schematic drawing has been added to the revised manuscript version (new Fig. 8).

In some cases also rewriting is needed, to expose the concepts in a clearer, more explicit manner (see as one example the discussion at page 10, lines 16-19).

Done.

Reference to models would also help understanding the interpretation of the data in support of a WRC-independent mechanism for the formation of lamellipodia.

Aside from the model in Fig. 8, we have also assembled a Supplementary Table (Table S1 in the revised manuscript) that summarizes all data obtained in a comprehensive fashion complementary to the schematic drawing (also requested by reviewer 2), so together, we think this has much improved the overall presentation of the data.

Figure 4 and S5, authors mention that VASP is an exception (page 10); I guess they refer to the fact that VASP is NOT accumulated at the cell edge (Figure 4); but then, VASP is clearly accumulated at adhesions at the cell edge in the same dKO clones (Figure S5). The conclusion at page 10 is not supported by the images shown in Figure S5. This is confusing and should be explained clearly.

We have reorganized the Figure a little bit due to other reviewer's requests, which is why the reference to Figures has slightly changed, but: Indeed, VASP is NOT accumulating as continuous line any more as it does at the edges of wildtype lamellipodia. This has now been specified more directly, in order to avoid potential confusion of reviewer and readers. The difficulty the reviewer is facing is that under certain conditions, such as in the classical Kate Nobes-type of experiment, in which high Rac and low Rho generates Rac-dependent focal complexes (as they were called back then), these align along the edges of quite narrow lamellipodia (see seminal focal complex-paper by the Hall lab from 1995, PMID: 7536630. In follow up work then with Alan Hall, we previously established that those focal complexes contain vinculin and are adhesive structures, but not drawn along forward with the protruding or lifting-up (i.e. ruffling) lamellipodium (Rottner et al., Curr Biol, 1999 PMID: 10375527). In addition though, those nascent adhesions also contained VASP (Rottner et al., 2001, PMID: 11598195), like any other type of adhesion, but only VASP (and not vinculin or zyxin) also accumulates at the very edges (back then called tips) of protruding lamellipodia (Rottner et al., 1999, Nat Cell Biol, PMID: 10559946). The difference between lamellipodia/LLS edge

© 2022. Published by The Company of Biologists under the terms of the Creative Commons Attribution License (https://creativecommons.org/licenses/by/4.0/).

accumulation of VASP and its accumulation in the rope of pearl- like arrangement of nascent adhesions is not always as easily distinguished we admit at the single cell level. However, the line scan analyses shown in Figs 4 and S6 clearly prove the point (n = 30 in each case), and with two Nap1/Hem1-KOs and one Sra1/PIR121 clone, we are displaying 3 independent examples of individual cells, in which this differential accumulation pattern can be consistently observed (Figs. 4 and S6). But this all makes sense, also considering the fact that WRC-subunits like Abi exclusively accumulate at protruding leading edges (see Stradal et al., 2001, PMID: 11516653).

We have also revised the text in corresponding section to make these differential VASP patterns clearer for future readers.

Considering also what authors describe in the case of VASP in cells overexpressing C.A. Cdc42 (Figure 5), it looks like authors refer to more subtle differences concerning the distribution of VASP at the edge (continuous line vs focal points).

This is exactly the point, and emphasized more clearly now in the revised manuscript text.

Moreover, in Figure 5 it is not sufficient to refer to a previous paper for control distribution of VASP: WT cells should be included in this figure as proper control from the same experiments as for KO clones.

The reason of using the C.A. GTPAse is not quite clear here. It is well known that C.A. GTPases may induce phenotypes deriving from an altered functional cycle, which is not physiological. What is the rationale for showing these experiments? and the relationship to what has been discussed earlier? This is not clear from the way the data are presented. And once more, a reference scheme would help.

We have now added a proper control of the Cdc42-CA expression in B16-F1 wildtype cells, as requested, which is now shown in new Fig. 5A (top panel). The rationale for using constitutively active Cdc42 in cells harbouring endogenous GTPases in transient, o/n transfections, is to potently trigger downstream signalling as alternative to growth factor stimulation, and as opposed to just increasing the amount of overall GTPase expression levels, which will likely not cause as robust effects as the use of constitutively active variants. Moreover, CA-Cdc42 is a well-established and useful tool to explore downstream effector function of the GTPase or cytoskeletal responses, such as signalling to Rac GTPase activation or triggering N-WASP-dependent actin assembly processes. Of note, the use of constitutively active Cdc42 also makes sense in the context of the established rise in Cdc42 activity levels upon treatment with the growth factors employed in previous experiments, and indeed, abolishment of growth factor- driven effects by genetic Cdc42 removal confirms this reasoning (Fig 6D). All this has now also been put into better context in corresponding text sections of the revised manuscript. As already mentioned above, the reference scheme and a comprehensive table summarizing all results are now provided in new Fig. 8 and Suppl. Table 1.

Reviewer #1 (Significance (Required)):

The study by Kage et al. addresses the molecular mechanisms required for the formation of Rac-dependent lamellipodia, by disrupting the subunit Nap1 and its paralogue Hem1 of the WRC complex. The authors identify in this way a pathway for the formation of Arp2/3- dependent lamellipodia-like structures that require Rho family GTPases but that are independent on WRC.

Thank you again, and agreed.

Reviewer #2

(Evidence, reproducibility and clarity (Required)):
The manuscript by Kage et al. describes a highly original study of novel lamellipodia-like structures (LLS), which are independent of the WAVE regulatory complex (WRC), in mouse melanoma and fibroblast cell lines in culture. First, the authors have observed that a KO of a key

© 2022. Published by The Company of Biologists under the terms of the Creative Commons Attribution License (https://creativecommons.org/licenses/by/4.0/).

WAVE subunit, NCKAP1, did not abolish the formation of lamellipodia in all clones they have obtained (an exact quantification is necessary there (1). They have concluded that an up-regulation of Hem1/NCKAP1L is responsible for the lamellipodia in some of their clones, and have indeed shown that Hem1 was present, by having inserted a GFP tag before Hem1 in the genome (2).

We thank the reviewer for the constructive comments, and have now added a quantification of differential lamellipodia formation in all NCKAP1-KO clones characterized (new Fig. S2).

The authors then moved on to inactivate both NCKAP1 and Hem1 in B16-F1 cells, and to study the cell motility and formation of protrusions. These cells, when starved and then stimulated with growth factors, were capable of making "smaller than lamellipodia" (3) LLSs. These structures were observed in all WRC-depleted cell lines, but not in RAC1-3 KO cells. The authors show that WAVE subunits were not present in LLS, though the data are not quite clear (4). Then, the expression of Cdc42 in double KO NCKAP1/Hem1 cells is shown to induce the LLS (5). Finally, the authors conclude that these ARP2/3 containing structures can exist in B16-F1 or NIH 3T3 mouse cells, independently of WRC, but in direct link with CdC42 and/or Rac stimulation (6).

We will hopefully clarify all specific questions below.

(1). In multiple experiments shown in the article, the occurrence of cells is described as "occasional" or "frequent". An exact quantification is necessary, to be able to evaluate these results. In each case of a "typical field", it will be very helpful to have the exact percentage of cells in question in a larger population.

This has now been provided in different quantifications, either on lamellipodia formation frequency in distinct Nap1-null clones (new Fig. S2), concerning frequency of LLS formation in distinct WRC-null clones (new Fig. 3A), as well as width (Fig. 3B) and F-actin intensity (Fig. 3C) in LLS.

(2) It is hardly a conventional method, to tag an endogenous protein by GFP in order to show its expression. Why did not a simple western blot work? At least a comment about the choice of this technique is necessary here. What is the level of Hem1 expression, compared to the original NCKAP1 expression? The GFP KI approach can help to answer this important question.

The answer is simple: the antibody was just not sensitive enough to see the protein by regular Western, not even in Nap1-KO clone #21, in which Hem1 expression was apparently upregulated. So a quantification as compared to wildtype by these direct means was not possible. However, although not quantitative, we were able to qualitatively and unambiguously show that Hem1 protein was increased in clone #21 as compared to B16-F1 wildtype, because we could detect the protein by co-IP with EGFP- tagged WRC subunits, as shown for Sra1 in Fig. S3A (right panel). This provides direct evidence for increased Hem1 expression in clone #21 as compared to wildtype B16-F1, and that this must derive from the endogenous Hem1 locus was confirmed by the EGFP knock-in approach (Fig. 1E). Precise quantitation of the difference would indeed require the generation of additional EGFP knock-in clones in the wildtype B16-F1 background, which would not, in our view, contribute any further to the conclusions of the paper, and was therefore considered beyond the scope of the current manuscript.

(3) A quantification is needed here, because it is not clear what the authors mean by these novel structures being "smaller" than the lamellipodia.

Here, the reviewer refers to LLS formation in WRC-null cells (point 3 in introductory text above): we have provided the requested quantification in new Fig. 3B/C.

(4) In Fig.4, upper panels, the quantification of ABI1 localisation rather gives an impression that ABI1 (and VASP, as well) is present in the cell body AND at the protrusions, contrary to other factors that are specifically enriched at the LLS edge, but not in the cell body. This does not mean that ABI1 and VASP are absent at the edge.

© 2022. Published by The Company of Biologists under the terms of the Creative Commons Attribution License (https://creativecommons.org/licenses/by/4.0/).

We respectfully disagree with the reviewer here. Previous Fig. 4 showed just the line scans of Abi at the top as compared to Cortactin in the same cells, and VASP one panel below. These line scans were re-positioned a little bit in the revised manuscript version, and can now be found (together with representative data images) in new Figures 4B and S6B. Note, that those line scans do not cover the cell body at all, but just measure relative protein amounts at the very cell edge (up to max. 2 µm from inside out, so the very cell edge). Thus, we are comparing relative accumulations in lamellipodia (see the peaks for Abi and cortactin in B16-F1 control), and still see peaks for cortactin or Arp2/3 complex, representing their accumulation or enrichment in the cell periphery, but no such peaks in WRC-deficient cells for Abi or VASP staining. We should emphasize that we are looking at relative protein amounts within each measurement, so normalized between 0 and 1 for each measurement, which makes them comparable from one cell to the next (n=30), and we are NOT comparing absolute intensities. So in case of no specific accumulation at the cell periphery, all you will see is a decrease of a homogenous signal at the cell periphery down to zero in regions outside the cell (i.e. at app. 2 µm and beyond on the right hand side of each graph), and no peak. And this will be the case irrespective of signal intensity in all measurements due to the normalization. Importantly, this is precisely what we see, peaks for Abi and VASP in controls (B16-F1), and a flat signal (due to normalization) falling down to zero in case of WRC-KOs. So it is the shape of the curves (peak *versus* no peak) that is relevant here!

In order to make this clear also to potential, future readers, we have added one sentence towards the end of the chapter describing line scan measurements in the Methods, which reads as follows:

"Due to this procedure, line scan measurements can only give relative information for each component concerning the distribution of fluorescent signal along the scan, but do not provide any quantitative comparisons between distinct components, neither in the same cells/measured regions nor in different cell types."

(5) It will be useful to clearly define "LLS", and also to quantify the frequency of their appearance in this experiment, as well.

This now refers to LLS formation upon active Cdc42 expression, point (5) in the corresponding introductory statement by the reviewer above. We have performed several quantitations of LLS formation frequency in completely independent experiments, and also done by different individuals, and the frequency is not very high, but ranges between app. 11 and 22% in WRC null-clones (see e.g. Fig. 6C/D/E and Supplementary table 1), which is very different from the frequency of lamellipodia seen upon Cdc42 expression in B16-F1 wildtype cells, which is virtually 100% (Fig. 6C).

(6). The interplay between the Rac family and Cdc42 in this study is completely unclear. The authors show that Rac KO cells never form the LLS, and that WRC-depleted cells form the LLS when overexpressing Cdc42. Can these factors compensate for each other? More experiments are necessary to illustrate this question, and a model will also be very useful, in order to fully understand the proposed hypothesis. It will also be very helpful if the authors could suggest a possible mechanism of Rac/CdC42-dependent mode of WRC-independent activation of ARP2/3 in the LLSs, at least as a model.

No, Rac and Cdc42 cannot compensate for each other, as LLS formation in the absence of WRC is abolished by both Rac removal and by Cdc42 removal. Please note that we are not showing here that in cells lacking the three Rac GTPases plus WRC (so called penta-KOs), LLS formation is also gone, because these structures are already eliminated in Rac-KOs alone, but we know that Penta-KOs cannot form LLS (unpublished data). In other words, the presence of any Rac GTPase is obligatory for LLS formation, and so is the presence of Cdc42. Furthermore, if we induce LLS formation by constitutively active Cdc42, LLS formation requires the presence of its downstream effector N-WASP. All this is clear and supported by additional experiments, as requested, for instance, by showing that Cdc42 has to directly interact with CRIB domain-containing effectors (such as N-WASP) in order to be able to stimulate LLS (see new Fig. 6E/F). The only point that has to remain open is how Rac signals to LLS formation, or in other words, why it is required for LLS formation. Answering this question requires future investigations.

© 2022. Published by The Company of Biologists under the terms of the Creative Commons Attribution License (https://creativecommons.org/licenses/by/4.0/).

We have attempted numerous things already to get more insight (as also specified below for reviewer 3), as we know already, for instance, that direct Rac binding to CRIB domain-containing effectors is not obligatory for the response. However, since we have hitherto not been able to identify the precise mode of action of Rac in the pathway, we have refrained from going into more detail in the revised manuscript, also not to complicate things further at this stage. However, the fact that we don't know how Rac is signaling to LLS, is clearly illustrated by a red arrow and question mark in our schematic in new Fig. 8!

Reviewer #2 (Significance (Required)):

This is a novel and interesting study, and I hope that it will be published soon. However, more precision, quantification and statistical analysis, and some additional experiments are needed, as well as some editorial work, in order to make the story clear and straightforward.

All done.

Referee Cross-commenting

I fully agree with the comments of Reviewer 1, especially with regard to the absence of a clear model for the proposed LLS protrusions (such model(s) should be included in the paper), and the contradictions concerning VASP localisation.

We hope to have clarified all those issues.

With regard to Reviewer 3, I do agree with the majority of the comments, except that I find that the manuscript represents potentially interesting and novel data, and not just a confirmation of findings from other groups. However, it is not ready to be published in its current form.

We thank reviewer 2 for confirming that our data are interesting and novel!

Reviewer #3 (Evidence, reproducibility and clarity (Required)):

Kage et al

In this study, the authors explore the types of actin protrusions that form in the absence of the Wave Regulatory complex (WRC). They conclude that small, Arp2/3 containing protrusions, that they call lamellipodia like structure, still form in cells where WRC components have been eliminated by CRISPR-Cas9-based gene knockout. These LLS structures require CDC42 and N-WASP. Somehow Rac is still required as well, but explanation for this result is given. Finally, there is a marked decrease in Ena/VASP localization to the leading edge upon WRC deletion. For the most part, the experiments use standard techniques and are well executed. The problem with this paper is not in the evidence presented, but rather that it is all just confirmatory of findings from other groups. Very little new insight into the formation of actin protrusions is presented (more on that below). A few specific issues with the data in the paper:

Naturally, we do not agree with these statements by the reviewer, and will explain the reasons for this more specifically below.

1. The VASP staining in the Nap/Hem knockout is not really completely missing from the leading edge, but rather reduced based on what I see in the images (Sup Fig 5). The WT cells have a much brighter band of staining, but this is likely a mixture of leading edge staining at the tip of the lamellipodia and a row of nascent adhesions just behind it.

The comment on VASP staining in Nap/Hem KO is not correct. We have provided several images (in different types/clones of WRC-KOs) plus line scan quantitations (see newly arranged Figs. 4, 5 and S6) that clearly show that the accumulation of VASP at the leading edge membrane is missing in WRC-KOs, as opposed to B16-F1 control. The reviewer is correct in the fact that VASP localization patterns in wildtype cells are quite complicated due to accumulation at lamellipodia edges, filopodia tips plus nascent and regular adhesions, all of the latter are still present (and quite abundantly in places) in WRC null cells. However, what's consistently

© 2022. Published by The Company of Biologists under the terms of the Creative Commons Attribution License (https://creativecommons.org/licenses/by/4.0/).

missing in the absence of WRC, is the very leading edge accumulation of VASP in a continuous line along LLS, which can still be prominently seen for other lamellipodial markers, such as lamellipodin (Lpd) and FMNL formins. All of this is also explained in much detail above, for instance for reviewer 1 (see specific discussion on Figure 4).

2. The significance of D21 in Hem1 is not clear to me. There is a known structure that could be consulted.

We agree that it would be worth looking into more detail why removal of D20 (we have corrected the position of the residue from D21 to D20) generates a null mutant, but interestingly, the residue and the sequence around it is identical in all murine Nap1 isoforms analyzed, strongly suggesting that the residue is important and sensitive to mutation. We will be happy to look into this topic in the future, but this is certainly beyond the scope of the current manuscript. Here, instead, we were exclusively interested in making sure that deletion of the respective aspartate - as has accidentally occurred in the low abundance allele present in Nap1/Hem1 KO #7 - generates a functional null mutant, which was experimentally proven in Fig. S5.

3. The Listeria experiments come completely out of the blue in the paper. They are not mentioned in the abstract, introduction or concluding remarks.

In spite of this comment, we think the *Listeria* data fit the manuscript perfectly well, for several reasons, the most relevant of which are:

(i) **Listeria entry into murine cells through InlB (InlA not functional in mouse) exclusively occurs through engagement of c-Met, the HGFR, and our LLS have been found to be triggered very efficiently by HGF as well (see above).**

(ii) **InlB-mediated Listeria invasion is quite well established to require Rac signalling and Arp2/3 complex-dependent actin remodelling, so it made a lot of sense to look at this physiologically relevant exploitation of c-Met signalling in the context of WRC complex function in fibroblasts, i.e. a distinct cell type. And indeed, the pathway turned out to functionally mimic all observations made above in a different cell type (B16-F1 melanoma) concerning actin-based protrusion, confirming that WRC is less relevant in actin remodelling downstream of Rac GTPases than the latter themselves (see Fig. 7 of the revised manuscript).**

In order to avoid the impression that the *Listeria* data come "out of the blue", we have now also mentioned them in both Abstract and Introduction; the concluding remarks have already referred to the data in the previous manuscript version (second last sentence).

4. The requirement for Rac, even in the absence of the WRC, is interesting, but the authors do nothing to explore this or even speculate about a possible mechanism. This was disappointing.

We have tried to use the revision time to dig more into the question why Rac is so important for this pathway and how it does it mechanistically, since its major downstream target, WRC, is missing in these cells. We are not showing all the data in the manuscript, but we have, for instance, performed rescue experiments with distinct Rac mutants in penta-KO cells (lacking WRC and on top all three Rac GTPases, as published by our lab previously, PMID: 31451035), and with simultaneous expression of constitutively active Cdc42. This allowed rescue of LLS formation in the context of distinct Rac versions (WT *versus* F37A *versus* Y40C, for instance). Surprisingly, both mutations reduced the response, but the C40 mutation did not eliminate it entirely, suggesting that Rac interactions with CRIB domain-containing mutants are not obligatory for the response, as also mentioned to reviewer 2 above. We are happy to provide these data to the reviewer and/or editor upon request. However, aside from established interactions with WRC through the D site of the latter (see PMID: 30393033), no other known effectors depending on F37 in Rac1 are known to date, so digging deeper into this question will require development of a completely new project.

© 2022. Published by The Company of Biologists under the terms of the Creative Commons Attribution License (https://creativecommons.org/licenses/by/4.0/).

Reviewer #3 (Significance (Required)):

This paper is not a very significant advance for the field (one in which I work directly).

We naturally disagree with this statement, and at least reviewer 2 does as well (see above)! I believe, we have already explained quite carefully on several occasions within this rebuttal letter and also in the manuscript, why we think our data are of high relevance and interest to the community.

For example, we know that Nap1 and Hem1 are functionally redundant proteins, therefore is not surprising that if you have a clone where you knockout Nap1 and Hem1 is upregulated that these cells would have proper lamellipodia.

Indeed, we admit that it was possible perhaps to anticipate from previous data that Nap1 and Hem1 might compensate for each other, but it was not clear to which extent *in vivo*, which we could clarify experimentally here! The answer being that at least concerning WRC-function in driving lamellipodia formation, Hem1 is capable of fully replacing Nap1, which is still quite surprising. In addition, it was unexpected for us that non- hematopoietic cells would be able to compensate for Nap1 removal with Hem1 upregulation of expression, since previously published Nap1 null fibroblasts did not seem to show this behaviour (PMID: 32646006). It is not clear at present where this difference comes from, but we will look after this in future studies.

We know that Ena/VASP proteins interact directly with WRC, therefore the reduction of localization of VASP upon WRC deletion is not surprising. Recent published work confirms this in C. elegans (PMID: 34281597).

The argument by the reviewer is not fair, because in mentioned *C. elegans* paper, the authors have only characterized the reduction of recruitment of an EVH1 domain- deficient version of the VASP-homologue UNC34 that abolishes interactions with all EVH1-ligands, and not just that with WRC, which is thus a quite different experiment. Yet, we have now cited this *C. elegans* work for completeness in our revised manuscript.

In any case, we have never implied in our manuscript (neither directly nor indirectly) that previous results have not already suggested Ena/VASP recruitment by WRC (see e.g. PMID: 28889942, but the argument is tricky in the complete absence of lamellipodia, since the accumulation of Ena/VASP in the lamellipodium requires the strict presence of such structures (see PMID: 10559946). This problem could be experimentally solved here by studying cells lacking WRC and stimulating them by various means. These cells still formed lamellipodia-like structures that harboured the entire actin assembly machineries tested (except for VASP). In this context, our study is providing the first, direct experimental situation, in which we can dissect out signalling requirements of specific factors in the lamellipodium from sole actin assembly/disassembly functions.

The upregulation of N-WASP to compensate for loss of Wave/Scar is known in other systems.

This is not a very specific comment, unfortunately, which is why it is a bit difficult for us to respond to it properly. However, we do not find evidence that N-WASP is upregulated in expression in Sra1/PIR121-KOs as compared to wildtype cells nor in Sra- 1/PIR121/Cdc42 triple KO cells (see Figs. 6B), so the statement by the reviewer in this report concerning N-WASP upregulation of expression is already incorrect! How and in which context N-WASP-dependent actin remodelling has been proposed to replace WRC-mediated actin assembly, has been mentioned on several occasions throughout the text and also in the Concluding Remarks.

We would like to thank all reviewers for their efforts reading our manuscript, and for constructive comments, all of which certainly improved the quality of the data shown and the clarity of the presentation.

© 2022. Published by The Company of Biologists under the terms of the Creative Commons Attribution License (https://creativecommons.org/licenses/by/4.0/).

Original submission

First decision letter

MS ID#: JOCES/2022/260364

MS TITLE: Lamellipodia-like actin networks in cells lacking WAVE Regulatory Complex

AUTHORS: Frieda Kage, Hermann Doering, Magdalena Mietkowska, Matthias Schaks, Franziska Gruener, Stephanie Stahnke, Anika Steffen, Mathias Muesken, Theresia E. B. Stradal, and Klemens Rottner
ARTICLE TYPE: Research Article

Based on your revisions and responses to the reviewers comments and questions from your original Review Commons submission I am happy to tell you that your manuscript has been accepted for publication in Journal of Cell Science, pending standard ethics checks.

© 2022. Published by The Company of Biologists under the terms of the Creative Commons Attribution License (https://creativecommons.org/licenses/by/4.0/).
